# Atherosclerotic Fibrous Plaques in Women Present ECM Remodeling Linked to TGF-β

**DOI:** 10.1161/CIRCRESAHA.125.327624

**Published:** 2026-02-05

**Authors:** Tim R. Sakkers, Eloi Mili, Hanna Winter, Daniek Kapteijn, R. Noah Perry, Nicolas Barbera, Kelsey Watts, Inês R. Dias, Denitsa Meteva, Marian Wesseling, Barend M. Mol, Gert J. de Borst, Dominique P.V. de Kleijn, Sander W. van der Laan, Mete Civelek, Stephen J. White, Lars Maegdefessel, Manuel Mayr, Gerard Pasterkamp, Michal Mokry, Ernest Diez Benavente, Hester M. den Ruijter

**Affiliations:** Laboratory of Experimental Cardiology (T.R.S., E.M., D.K., I.R.D., M. Mokry, E.D.B., H.M.d.R.), University Medical Center Utrecht, Utrecht University, the Netherlands.; Central Diagnostic Laboratory (M.W., S.W.v.d.L., G.P., M. Mokry), University Medical Center Utrecht, Utrecht University, the Netherlands.; Department of Vascular Surgery (B.M.M., G.J.d.B., D.P.V.d.K.), University Medical Center Utrecht, Utrecht University, the Netherlands.; Institute of Molecular Vascular Medicine, TUM Klinikum, Technical University Munich, Germany (H.W., L.M.).; German Center for Cardiovascular Research, Partner Site Munich Heart Alliance, Berlin (H.W., L.M.).; Department of Biomedical Engineering (R.N.P.), University of Virginia, Charlottesville.; Department of Genome Sciences (R.N.P., N.B., K.W.), University of Virginia, Charlottesville.; Charité-Universitätsmedizin Berlin, Corporate Member of Freie Universität Berlin and Humboldt-Universität zu Berlin, Germany (D.M.).; Department of Anesthesiology and Perioperative Medicine (M.C.), University of California, Los Angeles, CA.; Department of Human Genetics (M.C.), University of California, Los Angeles, CA.; Division of Cardiology, Department of Medicine (M.C.), University of California, Los Angeles, CA.; Institute for Precision Health, David Geffen School of Medicine (M.C.), University of California, Los Angeles, CA.; Faculty of Medical Sciences, Biosciences Institute, Newcastle University, United Kingdom (S.J.W.).; National Heart and Lung Institute, Imperial College London, United Kingdom (M. Mayr).; Division of Cardiology, Department of Internal Medicine II, Medical University of Vienna, Austria (M. Mayr).

**Keywords:** acute coronary syndrome, endothelial-mesenchymal transition, multiomics, plaque, atherosclerotic, sex differences

## Abstract

**BACKGROUND:**

Sex and atherosclerotic plaque histology are intertwined, with fibrous plaques being more prevalent in women. Plaque erosion, a significant contributor to acute coronary syndromes, is linked to fibrous plaques and is more prevalent in women than men. We hypothesize that the molecular drivers of histologically determined fibrous plaques differ between men and women.

**METHODS:**

Human end-stage atherosclerotic plaques were isolated from carotid endarterectomy patients included in the Athero-Express Biobank. Fibrous plaques were histologically assessed, linked to clinical characteristics, and processed for protein, bulk RNA, single-cell RNA, and DNA methylation data. We leveraged sex-differential gene expression and deconvolution analyses to uncover sex-biased molecular and cellular mechanisms. Spatial transcriptomics localized gene expression patterns in plaques. Furthermore, we studied the female-biased processes in human plaque endothelial cells and vascular smooth muscle cells stimulated with TGF-β (transforming growth factor-β), with or without SMAD3 (SMAD family member 3) inhibition.

**RESULTS:**

Of 1889 atherosclerotic plaques (1309 male and 580 female), fibrous lesions were observed in 50% of female and 31% of male patients. Compared with patients with atheromatous plaques (n=494), women with fibrous plaques exhibited a high prevalence of smoking, while men with fibrous plaques presented more often with diabetes. Female fibrous plaques were characterized by smooth muscle cell–driven ECM (extracellular matrix) remodeling, TGF-β response, and endothelial-to-mesenchymal transition, localized to the fibrous cap. Conversely, male plaques were linked to macrophage-mediated inflammation proximal to the core, dependent on diabetes. Finally, we experimentally confirmed these female-biased mechanisms, showing that TGF-β induced endothelial-to-mesenchymal transition in endothelial cells and ECM remodeling in vascular smooth muscle cells, both partly reversed by SMAD3 inhibition.

**CONCLUSIONS:**

Women and men with end-stage fibrous atherosclerotic plaques exhibit distinct clinical and molecular profiles. These mechanisms might be candidate pathways to understand plaque erosion from a molecular point of view and may provide promising targets for atherosclerosis therapies, as they account for both sex and plaque phenotype.

**Novelty and Significance**

**What Is Known?**

- Fibrous atherosclerotic plaques are more prevalent in women and are closely linked to plaque erosion, a major cause of acute coronary syndromes.
- Despite the clinical importance of investigating the mechanism of plaque erosion, symptomatic fibrous plaques are currently understudied.
- Fibrous plaques are considered structurally stable, yet they can become clinically dangerous through mechanisms involving endothelial dysfunction.
- Sex differences in gene regulatory networks suggest that women and men may develop fibrous plaque pathology through distinct cellular and molecular pathways.

**What New Information Does This Article Contribute?**

- Women with fibrous plaques showed a high prevalence of smoking, while men with fibrous plaques presented more often with diabetes.
- In women, fibrous plaques exhibited greater extracellular matrix remodeling and endothelial-to-mesenchymal transition, while plaques in men showed diabetes-related enrichment for inflammation.
- Spatial transcriptomics revealed distinct intraplaque localization: genes highly expressed in fibrous plaques from women were predominantly expressed in the fibrous cap and media, whereas those from men were concentrated in CD68^+^ macrophage–rich regions.
- These findings identify sex-biased molecular mechanisms that may represent promising targets to prevent plaque erosion on top of fibrous plaques.

Fibrous atherosclerotic plaques are common in women and play a central role in plaque erosion, a major cause of acute coronary syndromes whose prevalence appears to be rising. Despite the clinical importance of investigating the mechanism of plaque erosion, symptomatic fibrous plaques are currently understudied, and the biological processes that make these lesions dangerous remain poorly understood. This study addresses this gap by examining human end-stage fibrous plaques through integrated analyses of proteins, bulk and single-cell gene expression, DNA methylation, and spatial transcriptomics. We show that women with fibrous plaques are characterized by smooth muscle cell–driven extracellular matrix remodeling, TGF-β (transforming growth factor-β) signaling, and endothelial-to-mesenchymal transition concentrated in the fibrous cap. In contrast, fibrous plaques in men are more influenced by diabetes and characterized by macrophage-mediated inflammation near the plaque core. These sex-specific pathways were experimentally validated using primary human endothelial and vascular smooth muscle cells, where TGF-β induced the female-biased processes, and SMAD3 (SMAD family member 3) inhibition partially reversed them. Together, these findings reveal that fibrous plaques in women and men are biologically distinct and highlight molecular mechanisms that may underlie plaque erosion. Understanding these sex-dependent pathways offers new opportunities for developing targeted therapeutic strategies that account for both plaque phenotype and sex.

**Meet the First Author, see p e000748**

Plaque erosion significantly contributes to the incidence of acute coronary syndromes with a pronounced prevalence in women (51% versus 28% in men), especially among young women (<50 years: 77% versus 35% in men).^1^ Plaque rupture is more common in men (69% versus 37% in women) and is associated with elevated levels of cholesterol.^1^ In the current era of widely available lipid-lowering therapies, the proportion of acute coronary syndromes attributable to plaque erosion may be on the rise in both women and men.^2,3^ Plaque erosion is often linked to non–ST-segment–elevation myocardial infarction, in contrast to plaque rupture, which is more frequently the cause of ST-segment–elevation myocardial infarction.^2^

The mechanism of plaque erosion is thought to be endothelial dysfunction on top of fibrous plaques, which can trigger acute thrombosis.^4–7^ Women presenting with severe atherosclerotic disease more often present with fibrous plaques, while symptomatic fibrous plaques are also found in men, particularly young men.^8,9^ Fibrous plaques are considered stable plaques characterized by high collagen and smooth muscle cell (SMC) content. Yet, fibrous plaques can become symptomatic by mechanisms that promote plaque erosion. From a clinical perspective, diabetes is recognized as a pivotal risk for atherosclerosis, primarily linked to inflammation-driven unstable plaques prone to rupture.^10^ In contrast, smoking is another well-established risk factor for atherosclerosis and has been linked to plaque erosion in women.^1^ Notably, prolonged smoking leads to worse cardiovascular outcomes in women compared with men, but those mechanisms are yet to be fully understood.^11,12^ One potential mechanism could involve epigenetic regulation, as it has been shown that plaque methylation is influenced by smoking.^13^ Despite the clinical importance of investigating the mechanism of plaque erosion, symptomatic fibrous plaques are currently understudied.

As women more often present with fibrous atherosclerotic plaques, sex-stratified analyses of atherosclerosis have shed light on potential mechanisms of such plaques. We and others have shown that female atherosclerosis is characterized by gene regulatory networks distinct from those of men. These gene regulatory networks are active in both endothelial cell (EC) and SMC plasticity, including endothelial-to-mesenchymal transition (EndMT).^14,15^ EndMT is an important process in atherosclerosis where ECs acquire mesenchymal characteristics, leading to increased cellular migration, invasion, and ECM (extracellular matrix) production.^16,17^ Furthermore, these gene regulatory networks are enriched for estrogen receptor signaling, suggesting a role for sex hormones in the observed differences between female and male plaques.^14,15^ Sex and plaque phenotype are intimately linked and, therefore, difficult to disentangle. Moreover, the specific differences between sexes within the same plaque phenotype and the impact of associated risk factors remain unclear. Given the increasing prevalence of non–ST-segment–elevation myocardial infarction and fibrous atherosclerotic plaques over the past decades and their intrinsic link to sex,^2,3^ sex-specific studies may provide important novel insights into this critical part of plaque pathology. We hypothesized that sex differences exist within these fibrous lesions, with female fibrous plaques mainly driven by fibrotic processes, while male fibrous plaques are more influenced by immune-related mechanisms.

Therefore, we obtained human end-stage atherosclerotic plaques and specifically examined lesions classified by their histological features as fibrous. We studied risk factor profiles of these patients and explored sex differences within plaques at protein, gene, and DNA methylation levels. Spatial transcriptomics was used to locate the expression of these genes in situ in plaque tissue. We provide evidence that female fibrous plaques are characterized by SMC-driven ECM remodeling and EndMT located in the fibrous cap and media. Male fibrous plaques, on the other hand, have diabetes as an important risk factor and point to inflammation driven by CD68^+^ macrophages located in the intima.

## Methods

### Data Availability

Anonymized data and materials for the transcriptomics, epigenetic, and single-cell RNAseq have been made publicly available at DataverseNL and can be accessed at https://doi.org/10.34894/4IKE3T, https://doi.org/10.34894/TYHGEF, and https://doi.org/10.34894/D1MDKL. The scripts and other data sets that support the findings of this study are available from the corresponding author upon reasonable request.

### Study Population

The Athero-Express Biobank (AE) is an ongoing longitudinal biobank since 2002 aimed at investigating atherosclerotic plaques in patients undergoing carotid endarterectomy. All studies within the AE were conducted in accordance with the Declaration of Helsinki,^18^ and informed consent was provided by all study participants after approval for this study by the medical ethical committees of the 2 Dutch tertiary referral centers (UMC Utrecht and St. Antonius Hospital).

To identify plaque phenotype-specific properties, we focused our analysis on fibrous and atheromatous plaques (Figure S1A and S1B and excluding fibroatheromatous plaques), totaling 290 female and 416 male fibrous plaques and 91 female and 403 male atheromatous plaques. An overview of the number of plaques used for the different omics in this study is displayed in Figure S1A and S1B, the Table, and Tables S2, S4, S5, S12, S17, S22, and S23.

**Table.**
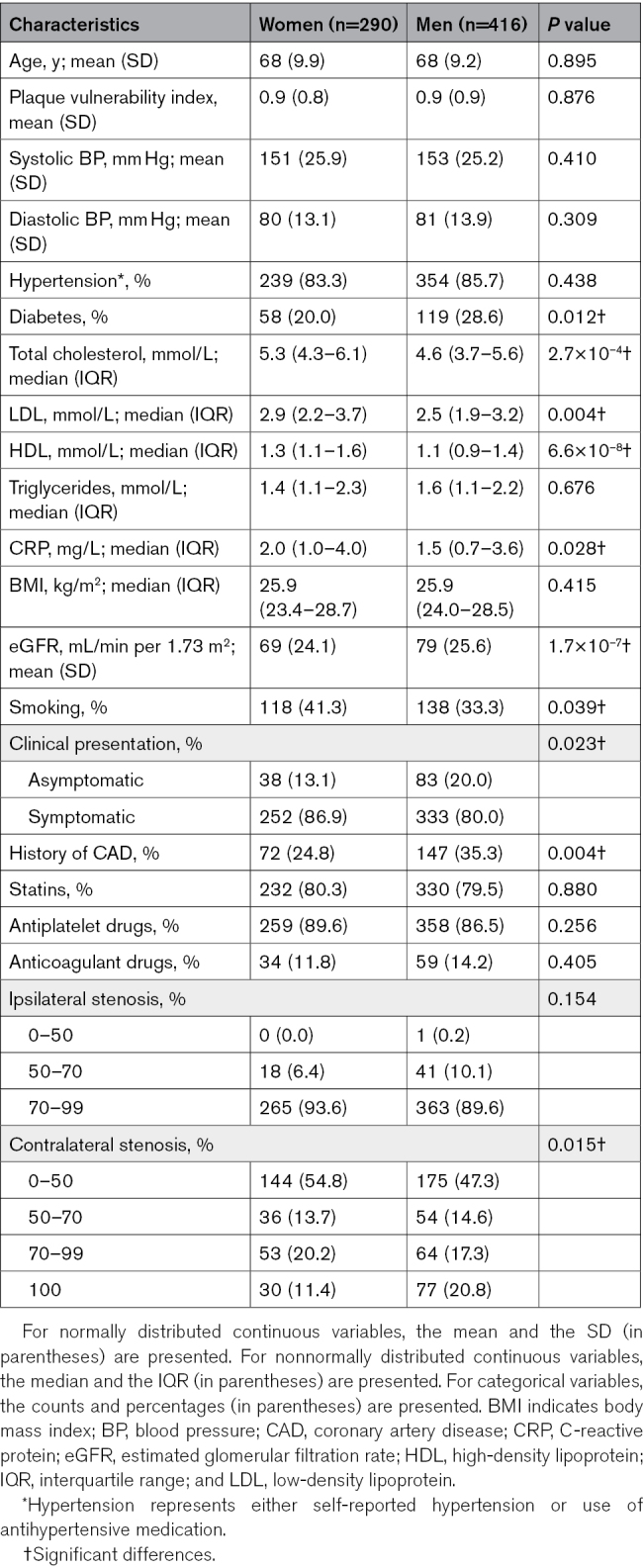
Clinical Characteristics and Risk Factor Profiles of 706 Atherosclerosis Patients With Fibrous Plaques

All methods regarding sample collection, plaque histology, bulk RNA sequencing, untargeted liquid chromatography–mass spectrometry proteomics, single-cell RNA sequencing, DNA methylation, and cell-type deconvolution are provided in the Supplemental Material (Supplemental Materials and Methods 1, 2, 4, 5, and 7 through 9).

### Statistical Analyses

All analyses were performed using R (version 3.6.2) and its IDE RStudio (version 1.2 and later). Baseline characteristics of the study population were summarized using the tableone package. Some cardiovascular risk factors were not always documented in the electronic health records and contained missing data, including total cholesterol, LDL (low-density lipoprotein), HDL (high-density lipoprotein), triglycerides, and CRP (C-reactive protein; Tables S1 and S3). Analyses were performed on the complete records for these variables. For categorical variables, the χ^2^ test was performed. Normality of continuous variables was assessed using the Shapiro-Wilk test. For normally distributed continuous variables, a 2-tailed unpaired Student *t* test was used, while, for nonnormally distributed continuous variables, this was a 2-tailed unpaired Mann-Whitney *U* test. Correlations were assessed using Pearson correlation coefficients. Differential expression analysis was performed using DESeq2, which fits negative binomial generalized linear models and applies Wald tests to evaluate the significance of model coefficients. *P* values were adjusted for multiple testing using the Benjamini-Hochberg false discovery rate. Gene Ontology and pathway enrichment analyses were performed using clusterProfiler, which applies hypergeometric testing for overrepresentation and Benjamini-Hochberg false discovery rate correction for multiple testing. The level of significance was set at *P*<0.05 for all tests.

To address potential effects of sex imbalances, we performed propensity score matching based on key atherosclerotic risk factors and clinical presentation. Further details on these analyses are provided in the Supplemental Material (Supplemental Materials and Methods 3).

### Spatial Transcriptomics of Human Carotid Plaques

Untargeted human carotid plaque samples for spatial transcriptomic analysis were derived from carotid endarterectomy and received from the Munich Vascular Biobank.^19^ Tissue sampling was performed following the declaration of Helsinki, with the approval of the local ethics committee (2799/10). Further details on sample preparation and analyses are provided in the Supplemental Material (Supplemental Materials and Methods 6).

### Human ECs for Transforming Growth Factor-β In Vitro Experiments

ECs were isolated from the luminal surface of human carotid plaques obtained from the AE (7 female and 11 male plaques; Table S24) and, along with human coronary artery ECs (Promocell, CAT C-12221; 2 female and 1 male donors), were used for in vitro experiments. Plaque ECs were characterized through microscopy, immunofluorescence staining, flow cytometry, bulk RNAseq, and monocyte adhesion assays under flow. ECs were stimulated with TGF-β (transforming growth factor-β), TNF-α (tumor necrosis factor-α), and SIS3 (specific inhibitor of SMAD family member 3) to evaluate gene expression changes. Detailed protocols for EC isolation, expansion, characterization, stimulation, and gene expression analyses are provided in the Supplemental Material (Supplemental Materials and Methods 10 through 16).

### Human Vascular SMCs for TGF-β In Vitro Experiments

Vascular SMCs (VSMCs) were isolated from ascending aortic explants obtained from 5 male and 5 female donors.^20^ VSMCs were stimulated with TGF-β and SIS3 to evaluate gene expression changes. Detailed protocols for VSMCs isolation, expansion, stimulation, and gene expression analyses are provided in the Supplemental Material (Supplemental Materials and Methods 17 and 18).

Please see the Major Resources Table in the Supplemental Material.

## Results

### Female and Male Patients With Fibrous Plaques Have a Distinct Risk Factor Profile

**Figure 1.**
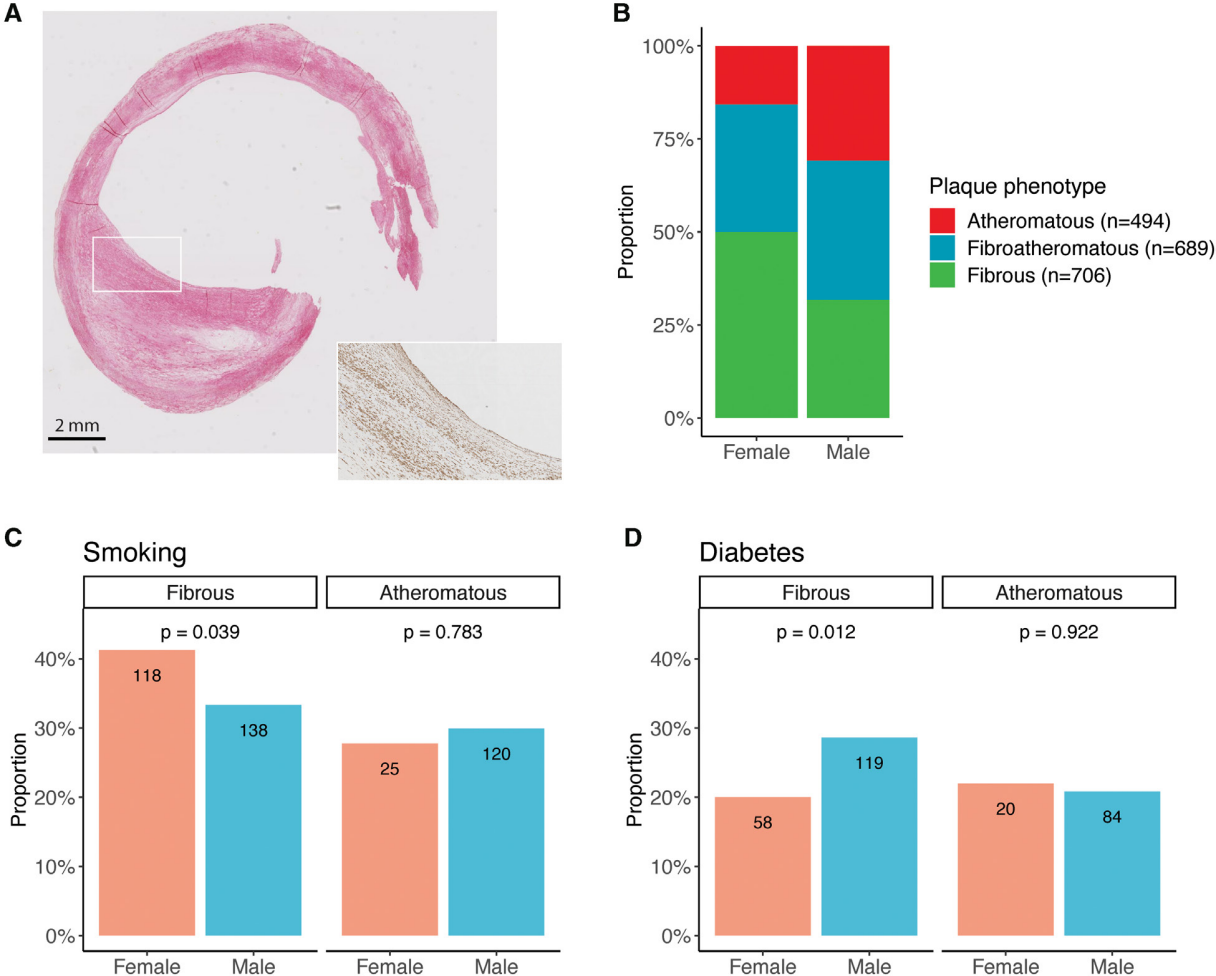
**Histological characterization and risk factor prevalence in patients with fibrous and atheromatous plaques in the Athero-Express Biobank.** Fibrous plaque with thick fibrous cap containing major staining for collagen (visualized by Picrosirius red staining; **left**) and smooth muscle cells (visualized by alpha-smooth muscle actin [α-SMA] staining; **right**). **A**, Representative fibrous plaque image displaying the characteristic morphology consistently observed across biological replicates. **B**, Distribution of plaque phenotypes in women and men who underwent carotid endarterectomy. The prevalence of smoking in women and men with fibrous plaques (**C**, **left**) vs atheromatous plaques (**C**, **right**). The prevalence of diabetes in women and men with fibrous plaques (**D**, **left**) vs atheromatous plaques (**D**, **right**). The numbers displayed within the bars indicate the number of patients in each group. Statistical test: χ^2^ test (**A**).

We selected all consecutive patients included in the AE for which histopathologic slides (Figure 1A) were available between 2002 and 2018 (n=1.889, 31% women). Histopathologic assessment revealed 50% of female and 32% of male plaques as being fibrous, and 16% and 31% as being atheromatous, respectively (Figure 1B). We analyzed 290 women and 416 men with fibrous plaques, both with an average age of 68 years (Table). In addition, we compared this cohort to 91 women and 403 men with atheromatous plaques with a mean age of 72 and 70 years, respectively (Tables S2 and S3). Plaque vulnerability index, which is a combined score of individual histological features (SMC content, collagen content, lipid content, and macrophage content),^15^ did not show differences between men and women (Table; Table S2). Significantly fewer women were asymptomatic compared with men (13% versus 20%; *P*=0.023). Women presenting with fibrous plaques exhibited a higher prevalence of smoking (41% versus 33%; *P*=0.039; Figure 1C), while men with fibrous plaques presented more often with diabetes (29% versus 20%; *P*=0.012; Figure 1D). These differences in risk factor prevalence were specific to patients with fibrous plaques and were not observed in patients with atheromatous plaques (Table S2). In addition, circulating cholesterol and inflammatory biomarkers were higher in women with fibrous plaques, while men presenting with fibrous plaques more often had a history of coronary artery disease and had higher estimated glomerular filtration rates (Table).

### Sex Differences in Gene Expression in Fibrous Plaques

Fibrous plaques, as defined histologically, may harbor distinct molecular mechanisms beyond their histological characteristics. Therefore, we recently investigated the transcriptomic landscape of atherosclerotic plaques and identified 5 plaque transcriptomic clusters, including the fibrocollagenous, intermediate, lipomatous, fibroinflammatory, and fibrocellular plaque phenotypes.^21^ We assigned a cluster to each of the plaques within the study based on overall gene expression patterns. In women, 64% of the fibrous plaques matched a fibrous-like phenotype (fibrocollagenous and fibrocellular), and 11% overlapped with an atheromatous-like phenotype (lipomatous and fibroinflammatory), while, in men, this was 50% and 23%, respectively (Figure 1B). A similar trend toward inflammatory clusters in men was observed when comparing atheromatous plaques of men and women (Figure S2C).

**Figure 2.**
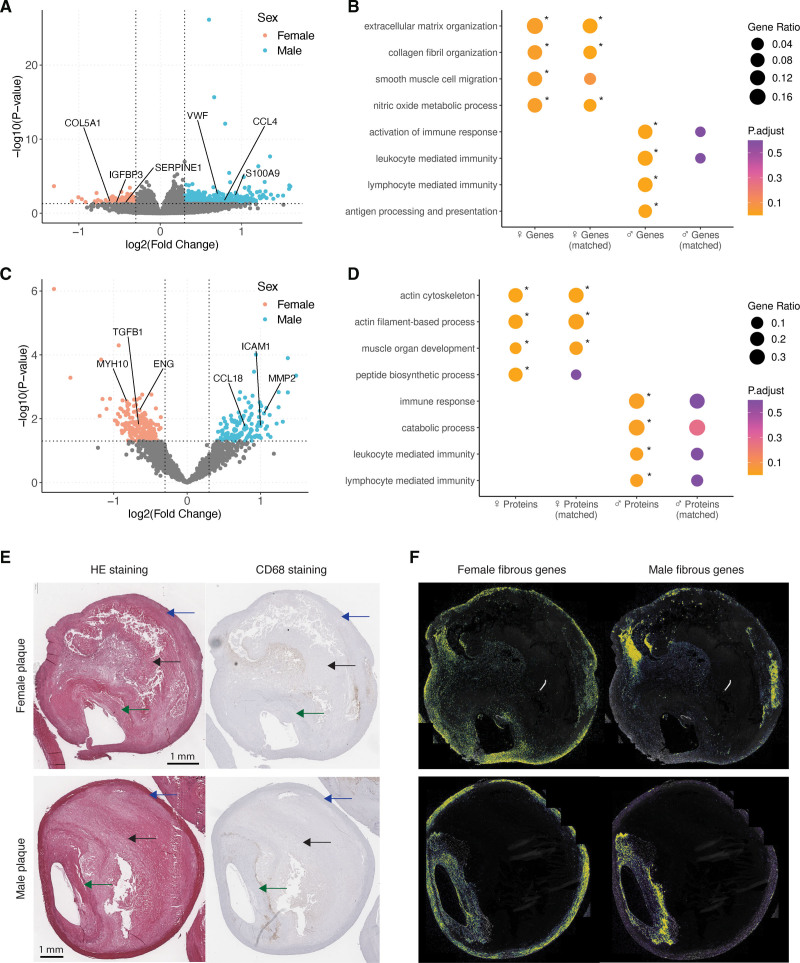
**Sex-specific gene and protein expression in fibrous plaques with spatial distribution. A**, A volcano plot is shown for the differential gene expression analysis between fibrous plaques from female and male patients. Gene Ontology (GO) enrichment analyses for female-biased (**B**, **left**) and male-biased genes (**B**, **right**) in fibrous plaques from the original and matched cohorts (*P_adjusted_<0.05). **C**, A volcano plot is shown for the differential protein abundance analysis between fibrous plaques from female and male patients. GO enrichment analyses for female-biased (**D**, **left**) and male-biased proteins (**D**, **right**) in fibrous plaques from the original and matched cohorts (*P_adjusted_<0.05). Hematoxylin–eosin (HE) (**E**, **left**) and CD68 (**E**, **right**) staining of female plaque (**top**) and male plaque (**bottom**) with fibrous cap (green arrow), core of the plaque (black arrow), and media (blue arrow). Density plots representing the spatial distribution of female gene candidates (**F**, **left**) and male gene candidates (**F**, **right**) in female plaque (**top**) and male plaque (**bottom**). Statistical tests: DESeq2 negative binomial generalized linear model+Wald test (**A** and **C**) and Hypergeometric test, Benjamini-Hochberg false discovery rate (**B** and **D**).

To elucidate the specific transcriptomic differences between male and female plaques, we performed sex-differential gene expression analyses on 191 fibrous and 187 atheromatous plaques (Table S5). A total of 20 902 protein-coding genes were measured in 61 female and 130 male fibrous plaques, and 29 and 158 atheromatous plaques, respectively. Differential expression analysis in the fibrous plaques identified 138 differentially expressed genes between the sexes. Of these, 32 were more highly expressed in female and 106 in male fibrous plaques (Figure 2A; Tables S6 and S7). Genes higher expressed in female fibrous plaques were enriched for ECM (*P*=1.9×10^−^^4^) and collagen fibril organization (*P*=1.7×10^−4^), SMC migration (*P*=3.2×10^−^^4^), and nitric oxide metabolic process (*P*=3.1×10^−^^4^; Figure 2B; Table S8). In addition, there were enrichment signals pointing to TGF-β signaling (*P*=2.2×10^−^^6^), EC activation (*P*=1.6×10^−^^5^), and EndMT (*P*=4.2×10^−^^13^), which were the top enrichments based on the BioPlanet, Elsevier Pathway Collection, and MSigDB Hallmark databases (Figure S2D through S2F; Table S9). The male fibrous genes were enriched for activation of immune response (*P*=4.1×10^−^^5^), leukocyte- and lymphocyte-mediated immunity (*P*=3.3×10^−^^6^), and antigen presentation (*P*=1.5×10^−^^6^; Figure 2B; Table S8), aligning with findings from the atheromatous plaque comparison (Figure S2G and S2H; Tables S10 and S11).

### Untargeted Plaque Proteomics Enriched for ECM Proteins Points to Smooth Muscle Biology and TGF-β Pathway in Female Fibrous Plaques

To understand the importance of ECM organization in fibrous plaques from women compared with men, we generated untargeted proteomics data enriched for ECM proteins using LC-MS (see the Methods section for details). Protein analysis was performed on 19 female and 46 male fibrous plaques, and 14 female and 46 male atheromatous plaques (Table S12). Differential abundance analysis in the fibrous plaques identified 343 differentially expressed proteins, of which 211 were higher expressed in female and 132 in male fibrous plaques (Figure 2C; Tables S6 and S13). Enrichment analyses linked female-biased proteins to cytoskeleton organization (*P*=2.6×10^−^^5^), actin filament–based movement (*P*=4.0×10^−^^5^), and muscle structure development (*P*=4.7×10^−^^6^; Figure 2D; Table S14), showing comparable patterns to the atheromatous plaque comparison (Figure S3A and S3B; Tables S15 and S16). Proteins more abundant in fibrous plaques from women include MYH10 (myosin heavy chain 10), ACTN4 (alpha-actin 4), ENG (endoglin), and TGFβ1 (Figure S3C). ACTN4 primarily functions in cytoskeletal organization and cellular junctions, structural changes important for cell detachment and motility.^22^ Similar to ACTN4, MYH10 is involved in cellular contractility and motility.^23^ ENG is a coreceptor that enhances TGFβ1 signaling, one of the most important stimuli for EndMT.^16,24^ The male-biased proteins were enriched for immune response (*P*=2.0×10^−^^4^), leukocyte- and lymphocyte-mediated immunity (*P*=7.1×10^−^^5^), and catabolic processes (*P*=1.8×10^−^^4^; Figure 2D; Table S14). Proteins more abundant in fibrous plaques from men include CCL18 (C-C motif chemokine ligand 18), ICAM-1 (intercellular adhesion molecule-1), CXCL16 (C-X-C motif chemokine ligand 16), and MMP2 (matrix metallopeptidase 2; Figure S3D). CCL18 is a chemokine that contributes to the recruitment of immune cells to the site of plaque formation and has been linked to plaque instability through macrophages.^25^ ICAM-1 facilitates the adhesion of leukocytes to ECs, promoting their migration into the vascular wall.^26,27^ CXCL16 is a chemokine that mediates the recruitment of these leukocytes to the plaque site and is involved in the uptake of oxLDL (oxidized low-density lipoprotein) by macrophages.^28^ Finally, MMP2 is involved in the breakdown of ECM and is associated with unstable coronary artery disease.^29^

### Sensitivity Analyses on Plaque Transcriptomics and Proteomics in the Propensity-Matched Cohorts

To account for the potential impact of imbalanced sex distributions in our cohort, we repeated the transcriptomic and proteomic analysis on propensity-matched cohorts (Tables S17 through S21) with propensity scores based on key atherosclerotic risk factors, including age, hypertension, smoking, and diabetes (Figure S4A and S4C). While the female-specific signals were retained, the immune signal observed in male plaques was not significant after matching in both genes and proteins (Figure 2B and 2D). To further investigate this, we adjusted for risk factors in the original transcriptomic cohort using a leave-one-out approach. This revealed that the sex differences in immune processes were dependent on diabetes (Figure S5), suggesting that the immune differences are related to diabetes prevalence in men compared with women.

In addition to the risk factor-matched cohorts (Figure S4A and S4C), we created propensity-matched cohorts based on clinical presentation (Figure S4E and S4G). These analyses demonstrated strong correlations in effect sizes for all identified genes (mean correlation=0.85 and 0.9; *P*=1.73×10^−^^40^ and 6.97×10^−^^50^; Figure S4B and S4F) and proteins (mean correlation=0.97 and 0.97; *P*=5.07×10^−^^201^ and 1.44×10^−^^211^; Figure S4D and S4H) between the original and matched cohorts.

We also performed a transcriptomic analysis stratified by statin use, demonstrating strong correlations in effect sizes with the original findings for both (correlation statin users: 0.93; *P*=5.68×10^−^^61^, correlation nonusers: 0.63; *P*=1.36×10^−^^16^).

### Spatial Transcriptomics Localizes Expression of Female Enriched Genes to the Fibrous Cap and Media, and Male Ones to CD68^+^ Macrophage Clusters

Spatial transcriptomics provides a powerful approach to studying the spatial distribution of gene expression within the plaque tissue microenvironment. To investigate these distributions, we performed spatial transcriptomics on 1 female plaque and 1 male plaque (from the Munich Vascular Biobank^30^; see Supplemental Materials and Methods 6), both exhibiting a clear fibrous cap, lipid core, media (hematoxylin–eosin stainings; Figure 2E), and clusters of macrophages (CD68 staining; Figure 2E). A panel of 26 gene candidates was selected, derived from our transcriptomic and proteomic analyses, in combination with the spatial transcriptomics data. This selection consisted of 13 female-biased and 13 male-biased fibrous genes (individual plots available in Figures S6 and S7).

Female fibrous genes showed high expression in the fibrous cap near the carotid artery lumen and within the media (Figure 2F, left), areas characterized by abundant collagen and SMCs. In contrast, male fibrous genes exhibited expression in regions distal to the lumen, colocalizing with macrophage-rich clusters (Figure 2F, right). These findings suggest sex-specific spatial gene expression patterns in fibrous plaques. These patterns align with the transcriptomic and proteomic findings of sex-specific enrichment for ECM organization, TGF-β and EndMT processes in female plaques, and immune and inflammatory responses in male plaques.

### Single-Cell RNA Sequencing Expression of Sex-Biased Genes Points to SMCs, ECs, and Macrophages

**Figure 3.**
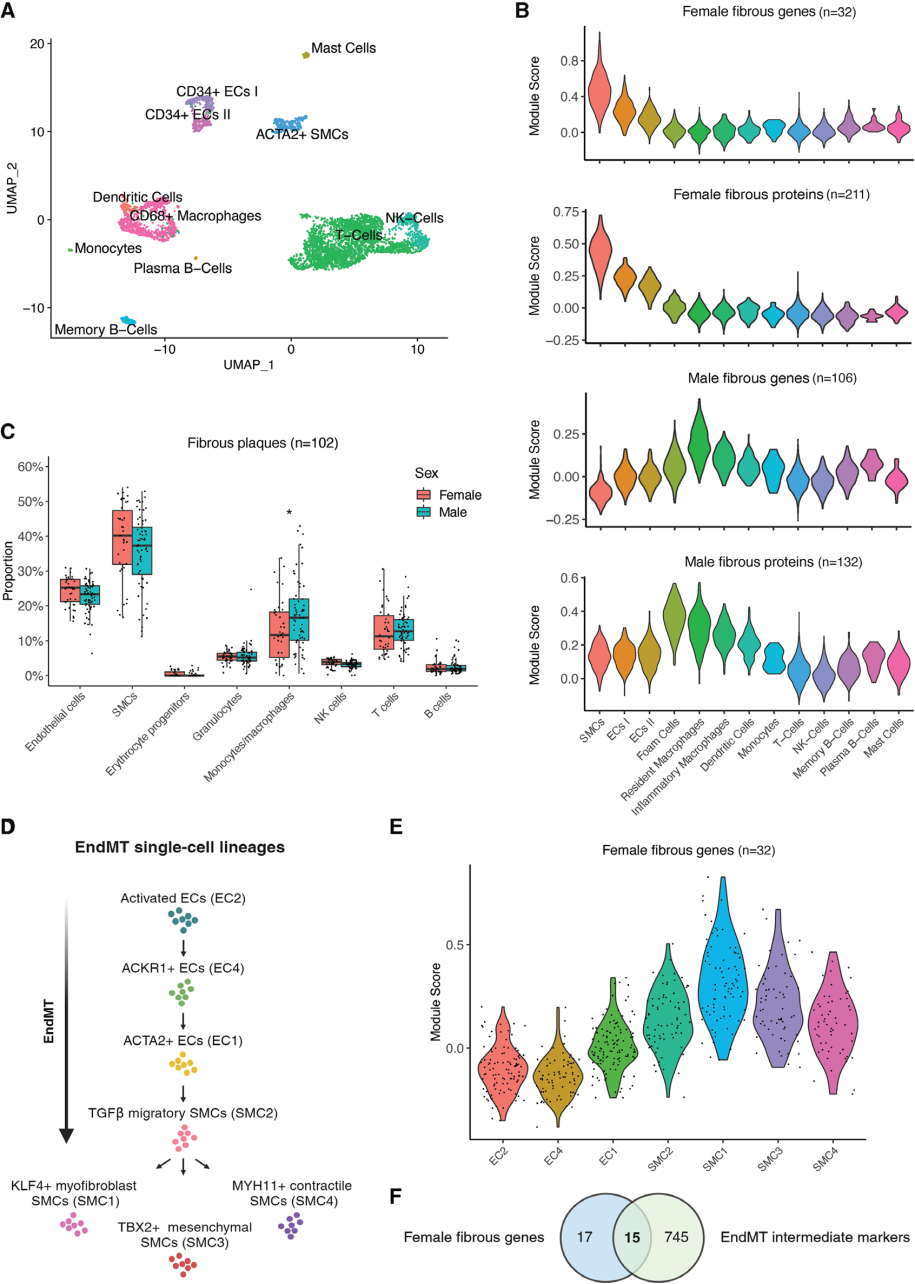
**Single-cell and epigenetic insights into endothelial and smooth muscle cell contributions to female fibrous plaques. A**, Uniform Manifold Approximation and Projection (UMAP) plot of 4948 single cells from carotid plaques (20 female and 26 male patients). Module score (see the Methods section) expression of female fibrous genes and proteins (upper 2 figures, **B**) and male fibrous genes and proteins in plaque cell types (lower 2 figures, **B**). Boxplot is shown for the sex-stratified deconvolution analysis on fibrous plaques using DNA methylation data. **C**, Proportion represents the predicted contribution (%) of each cell type to the fibrous plaque. **D**, Identification of human endothelial-to-mesenchymal transition (EndMT) single-cell lineages in plaque endothelial cells (ECs) and smooth muscle cells (SMCs) using pseudotime analysis.^32^ **E**, Violin plot showing the expression of female fibrous genes along the trajectory of EndMT lineages. **F**, Overlap of female fibrous genes with previously identified EndMT intermediate markers. Statistical test: 2-tailed unpaired *t* test (**C**).

To assess which cell types underlie the observed sex differences in gene expression and protein abundance, we analyzed single-cell RNA sequencing (scRNAseq) data from carotid plaques of an independent subset of 46 patients from the same cohort (AE: 20 women and 26 men; Figure 3A; Table S22). Genes upregulated in female fibrous plaques were highest expressed in SMCs, while also showing high expression in ECs (Figure 3B). In contrast, genes upregulated in male fibrous plaques showed the highest expression in resident macrophages and inflammatory macrophages. scRNAseq data mapped the proteins more abundant in female fibrous plaques to SMC and EC phenotypes. In contrast, the proteins more abundant in male fibrous plaques were highest expressed in foam cells, resident macrophages, and inflammatory macrophages (Figure 3B). We performed this analysis stratified by whether patients were taking statins at the time of the endarterectomy and found no differences between patients prescribed statins and those not prescribed statins (Figure S8A and S8B). In general, the cell-type–specific expression from plaque proteomics closely mimicked that found in plaque transcriptomics, which was also seen for the female genes and proteins in the atheromatous comparison (Figure S9A and S9B). To assess whether these differences were specific to differential expression in specific cell types, we assessed the module score expression of our genes of interest at the single-cell level. Female fibrous genes did not reach statistical significance but showed a trend toward higher expression in female SMCs and ECs (*P*=0.088 and *P*=0.085, respectively; Figure S10A). Proteins enriched in female fibrous plaques were significantly higher in female ECs (*P*=0.016; Figure S10B). In contrast, male fibrous genes and proteins showed consistently higher expression in all cell types from female donors (all *P*<0.001), except for male fibrous genes in SMCs (*P*=0.27; Figure S10C and S10D).

### Cell-Type Deconvolution Based on DNA Methylation Supports a Different Ratio of Immune Cells/SMC+ECs Between Male and Female Fibrous Plaques

To study if sex differences in cell-type-specific expression can be linked to differences in cellular composition, we performed deconvolution analyses on plaque DNA methylation data (Table S23). A comprehensive DNA methylation atlas comprising 39 cell types sorted from 205 healthy tissue samples^31^ underwent filtering to select cells expected to be present in atherosclerotic plaques (Supplemental Materials and Methods 9; Figure S11). This condensed methylation atlas, consisting of 8 selected cell types, served as a reference to estimate the cellular composition of 102 fibrous (Figure 3C) and 117 atheromatous plaques (Figure S9C). These results indicate that the cellular landscape in the fibrous plaque mainly consists of SMCs (37%), ECs (23%), macrophages/monocytes (16%), T cells (13%), and granulocytes (6%). Male fibrous plaques contained significantly more macrophages and monocytes compared with their female counterparts (mean difference=4.5%; *P*=0.022). Although not significant, there appears to be a trend toward higher proportions of SMCs and ECs in female plaques. Deconvolution of bulk RNAseq data from 191 fibrous and 187 atheromatous plaques, using scRNAseq-defined clusters as reference, supported these findings, showing increased macrophage content in male fibrous plaques, particularly resident (mean difference=3.0%; *P*=0.011) and inflammatory subtypes (mean difference=0.4%; *P*=0.020; Figure S12A and S12B). Male plaques also exhibited significantly more B cells (mean difference=0.4%; *P*=0.022), while female plaques showed a trend toward higher SMC proportions. Collectively, these analyses identify SMCs, macrophages, and ECs as important players for inherent sex disparities in plaque transcriptomics and proteomics.

### Female Fibrous Gene Patterns Point to TGF-β Signaling and EndMT

Enrichment analyses highlight TGF-β signaling as one of the top pathways in female fibrous plaques, alongside EndMT (Figure S2D and S2F). Key proteins involved in these pathways, including TGFB1 and ENG, were significantly more abundant in female fibrous plaques compared with male plaques (Figure S3C). In addition, our deconvolution analysis of methylation data suggests an increased presence of ECs and SMCs in female fibrous plaques (Figure 3C). Given that cellular transitions within atherosclerotic plaques are known to contribute to plaque progression, particularly in female plaques,^14–17^ we hypothesize that TGF-β–induced EndMT, which involves both EC and SMC signals, may explain the observed sex differences.

We used EndMT single-cell lineages previously defined through pseudotime trajectory analysis of the same human carotid plaque scRNAseq data set described above.^32^ That study identified 3 EndoMT trajectories linking endothelial and SMC subpopulations. Lineages were validated in vivo using lineage-traced apolipoprotein E–deficient mouse plaques (endothelial cell–specific, tamoxifen-inducible Cre recombinase [Cdh5-CreERT2] crossed with a Rosa26-eYFP reporter mice), demonstrating consistency with human data. Intermediate clusters along the trajectory, labeled EC1 and SMC2, were identified based on their pseudotime positions and characteristic marker gene expression (Figure 3D).

EC1 cells are positive for the endothelial marker CD34 and the SMC marker ACTA2, with differential genes enriched for TGF-β response, SMC proliferation, and EndMT. SMC2 cells express markers indicative of cellular migration, with differential genes enriched for TGF-β and bone morphogenetic protein signaling.^15^ Using these established lineages, we analyzed the expression of the female-biased fibrous genes. The expression of these genes along the lineages revealed increasing expression consistent with the direction of the EndMT lineages (Figure 3E). Notably, nearly half of the female fibrous genes (15 of 32, referred to as female candidate genes) overlapped with the identified intermediate markers for mid-stage EndMT (Figure 3F), showing increased expression in EC1 and SMC2 cells (Figure S9D). Because EndMT is a well-established driver of vascular calcification,^33–35^ we studied whether the expression levels of the 15 female candidate genes correlated with the extent of calcification within plaques. In female plaques, the expression of these genes showed a significant positive association with calcification severity (Figure S13A). In contrast, we did not find this correlation in male plaques.

Smoking is associated with fibrous plaques in women (Figure 1C) and can have strong effects on DNA methylation in plaque tissue^13^ (Figure S13B). To investigate the effect of smoking on the regulation of the female candidate genes, we obtained DNA methylation data from 38 female and 64 male fibrous plaques. We specifically examined cytosine-phosphate-guanine dinucleotides (CpGs) in the promoter regions (transcription start site ≤1 kb) of the 15 female candidate genes, resulting in 9 promoter CpGs associated with 4 genes (3 CpGs with *COL5A1*, 2 CpGs with *IGFBP3*, 2 CpGs with *PCOLCE2*, and 2 CpGs with *FSTL1*). We found that these promoter regions are significantly hypomethylated in women who smoke and have fibrous plaques compared with women with fibrous plaques who do not smoke (*P*=0.02; Figure S13C). Upon closer examination by gene, the promoter region of *IGFBP3* was significantly hypomethylated (cg20592075 and cg25840173; Figure S13D). *IGFBP3* is a genome-wide association study-identified risk locus for coronary artery calcification^36^ and a mesenchymal marker indicative of EndMT. This hypomethylation was not observed in male plaques.

### TGF-β Stimulation of Human Plaque ECs Increases Female Candidate Gene Expression

**Figure 4.**
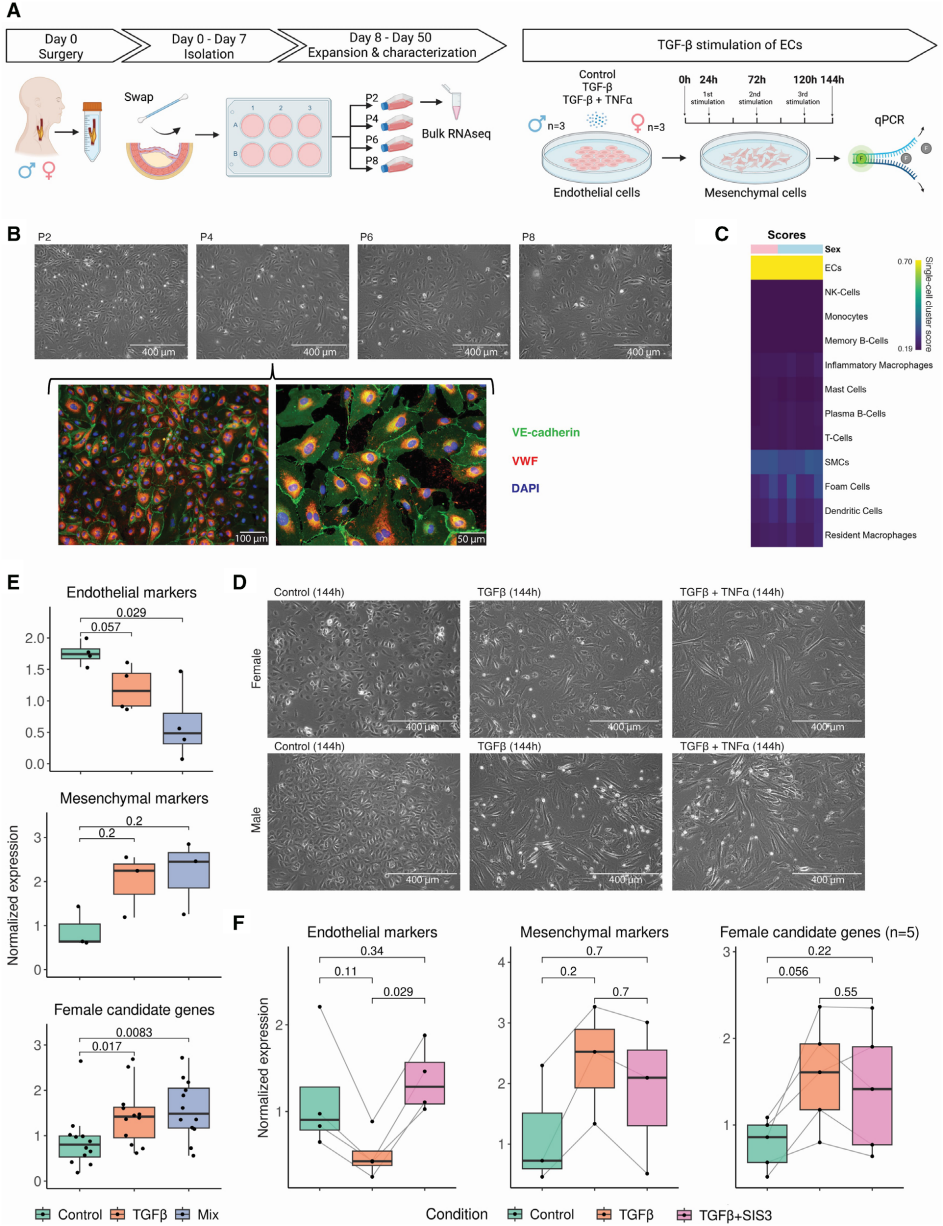
**Isolation and TGF-β (transforming growth factor-β) stimulation of human primary plaque endothelial cells. A**, Overview of plaque endothelial cell (EC) isolation and experimental design for TGF-β stimulation of ECs (see the Methods section). Representative microscopy images of plaque ECs from passage 2 to passage 8, along with fluorescence images of plaque ECs from passage 4 stained for VE-cadherin (vascular endothelial cadherin; green), VWF (von Willebrand Factor) (red), and nuclei (4′,6-diamidino-2-phenylindole [DAPI], blue; **B**). **C**, Heatmap displaying the bulk RNA expression of cultured plaque ECs mapped to single-cell RNAseq from plaques. Representative microscopy images of female (**D**, **top**) and male (**D**, **bottom**) plaque ECs, shown unstimulated, stimulated with TGF-β, or stimulated with TGF-β+TNF-α (tumor necrosis factor-α) after 144 hours. Boxplots showing changes in normalized quantitative PCR expression of known endothelial markers (**E**, **top**) and mesenchymal markers (**E**, **middle**) compared with control (=no stimulation), alongside the female candidate genes (**E**, **bottom**), across different conditions; mix=TGF-β+TNF-α. Boxplots showing changes in normalized quantitative PCR expression of known endothelial markers (**F**, **left**) and mesenchymal markers (**F**, **middle**) compared with control (=no stimulation), alongside female candidate genes (n=5; **F**, **right**), stimulated with TGF-β with or without SMAD3 (SMAD family member 3) inhibition by SIS3 (specific inhibitor of SMAD3). Statistical test: 2-tailed unpaired Mann-Whitney *U* test (**E** and **F**).

We used primary plaque ECs (6 female and 11 male; overview in Table S24) to further understand the interplay between TGF-β signaling, ECs, and the female candidate genes. Plaque ECs were isolated from the luminal surface of human atherosclerotic plaques (3 female and 3 male) and stimulated with TGF-β to evaluate the expression of the 15 female candidate genes of interest. Endothelial markers and mesenchymal markers were used as positive controls for the TGF-β stimulation experiment. The isolation process involved swabbing the plaque lumen with a digestive enzyme solution, followed by in vitro culture of these cells (Figure 4A; see the Methods section). The isolated plaque ECs consistently exhibited an endothelial-like cobblestone morphology across multiple passages and expressed hallmark endothelial markers, including von Willebrand Factor localized within the cytoplasm near the nucleus and VE-cadherin (vascular endothelial cadherin) at the transmembrane (Figure 4B). These plaque ECs were further characterized through flow cytometry (n=6), revealing cell marker expression profiles consistent with EC identity (Figure S14A and S15). In addition, to validate their EC identity, bulk RNA sequencing was performed on the plaque ECs at passage 2. Mapping the expression of cultured plaque ECs to single-cell RNAseq from plaques (Figure 3A) confirmed that the swabbed plaque cells were similar to the EC population of plaque-derived single cells (Figure 4C). To validate the maintenance of sex-chromosome complement in this model and, therefore, their genetic sex complement, we identified transcriptomic differences between male and female plaque ECs (Figure S14B). We confirmed the genetic sex of the cells through the sex-chromosome genes (ie, *XIST* in female ECs and *UTY* in males ECs). In a second analysis, we removed the sex chromosomes to focus on autosomal expression differences. Genes upregulated in female ECs were significantly enriched for pathways related to metabolism and mitochondrial processes, whereas male-biased genes showed enrichment for inflammation and epigenetic regulation (Figure S14C), suggesting that part of the variation observed in plaque composition can be linked to cell type–specific sex differences.

Functionally, the isolated plaque ECs showed increased expression of adhesion molecules at the surface and supported monocyte binding under flow. Fluorescence-activated cell sorter analysis on plaque ECs from 6 donors (2 female and 4 male) showed significant increases in VCAM-1 (vascular cell adhesion molecule-1) and P-selectin surface expression after TNF-α or combined TGF-β+TNF-α (mix) stimulation at both 24 and 144 hours, while ICAM-1 remained unchanged (Figure S16A and S16B). E-selectin levels were largely unaffected, with only a decrease observed under mix stimulation at 24 hours (Figure S16A). No changes in surface markers were observed after TGF-β stimulation alone. Using ibidi µ-Slides, monocyte adhesion under flow was measured after 5 hours of TNF-α stimulation in plaque ECs from 6 donors (3 female and 3 male), demonstrating increased monocyte binding without sex-specific differences (Figure S16C through S16F).

As enrichment analysis in our study highlighted TGF-β signaling as one of the top pathways in female fibrous plaques, we studied whether the expression of the female candidate genes was changed upon TGF-β stimulation. Isolated plaque ECs were stimulated under 2 conditions: TGF-β alone (10 ng/mL) and a combination of TGF-β (10 ng/mL) with TNF-α (25 ng/mL) as described previously.^32^ These stimulations induced pronounced morphological changes, with cells adopting an elongated shape, typical of mesenchymal cells, particularly visible under the combined TGF-β and TNF-α treatment (Figure 4D). Analysis of quantitative PCR expression data for EC markers (*PECAM1*, *CD34*, *VWF*, and *VE-cadherin*) was found to be downregulated upon TGF-β and TNF-α stimulation compared with control (Figure 4E), while expression of the mesenchymal markers (*FN1*, *Cadherin-2*, and *TAGLN*) showed an increasing trend although statistical significance was not achieved (Figure 4E). Notably, TGF-β stimulation significantly increased the expression of the female candidate genes compared with control (mean difference=0.81; *P*=0.017) and combined with TNF-α (mean difference=0.89; *P*=0.008; Figure 4E). Sex-stratified analyses are displayed in Figure S17B through S17D. Similar results were found in commercially available human coronary artery ECs (3 donors, 2 males and 1 female; Figure S17E and S17F). To evaluate whether downstream inhibition of TGF-β signaling could reverse its transcriptional effects in plaque ECs, we performed an additional TGF-β experiment with SIS3, a selective SMAD3 (SMAD family member 3) inhibitor. Quantitative PCR analysis showed a downward trend for endothelial markers compared with control and an upward trend for mesenchymal markers (Figure 4F), consistent with the direction of effects previously observed upon TGF-β stimulation (Figure 4E). However, these trends were partially attenuated by SIS3 cotreatment, suggesting partial mitigation of EndMT via SMAD3 inhibition. TGF-β also induced an upward trend for the female candidate genes, and this effect was still seen with SIS3 treatment although *SERPINE1, PCOLCE1*, and *FSTL1* showed signs of SMAD3-dependent inhibition (Figure 4F).

### TGF-β Stimulation of VSMCs Increases ECM Genes

**Figure 5.**
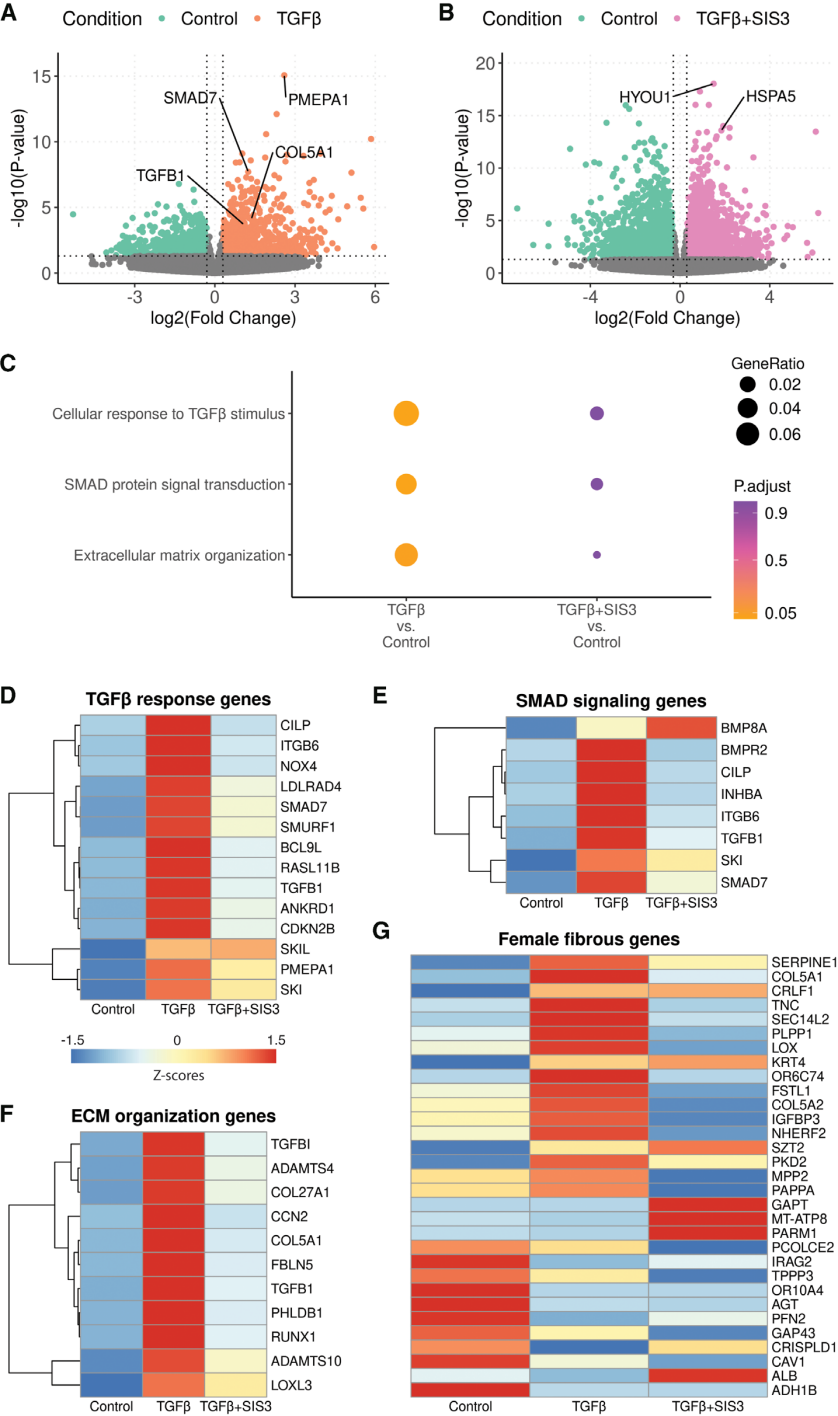
**TGF-β (transforming growth factor-β) stimulation of human primary vascular smooth muscle cells (VSMCs).** Volcano plot of differential gene expression of VSMCs stimulated with TGF-β (**A**) or TGF-β+SIS3 (specific inhibitor of SMAD family member 3; **B**) compared with control (unstimulated). Gene Ontology (GO) enrichment analyses for genes induced by TGF-β (**C**, **left**) and TGF-β+SIS3 (**C**, **right**) in VSMCs (*P_adjusted_<0.05). Heatmaps show the expression patterns of TGF-β response genes (**D**), SMAD3 (SMAD family member 3) signaling genes (**E**), ECM (extracellular matrix) organization genes (**F**), and female fibrous plaque–associated genes (**G**) upon TGF-β stimulation, with (**right**) or without (**middle**) SMAD3 inhibition by SIS3. Heatmaps display row-scaled Z-scores (per gene), with values standardized across conditions. Statistical tests: DESeq2 negative binomial generalized linear model+Wald test, Benjamini-Hochberg false discovery rate (BH-FDR; **A** and **B**), and hypergeometric test, BH-FDR (**C**).

We investigated the contribution of VSMCs to TGF-β–driven ECM remodeling using primary human aortic VSMCs (5 female and 5 male). Cells were stimulated with TGF-β, with or without SMAD3 inhibition by SIS3. Differential expression analysis identified 1749 genes differentially expressed between TGF-β–stimulated and control VSMCs (853 upregulated and 896 downregulated; Figure 5A; Table S26) and 3807 genes between SIS3-treated and control VSMCs (1531 upregulated and 2276 downregulated; Figure 5B; Table S27). TGF-β stimulation induced strong upregulation of all genes enriched for TGF-β response, SMAD signaling, and ECM organization (*P*_adjusted_<0.05; Figure 5C; Tables S28 and S29), which was not seen with cotreatment by SIS3. Specifically, 8 of 14 TGF-β response genes, 5 of 8 SMAD signaling genes, and 10 of 11 ECM organization genes were significantly upregulated by TGF-β (*P*_adjusted_<0.05; Figure 5D through 5F; Figure S18A through S18C), but not with SIS3, confirming SMAD3-dependent regulation. As ECM remodeling was identified as one of the main processes in female fibrous plaques, we next performed a sex-stratified analysis on ECM organization genes. Both female and male VSMCs displayed increased expression of ECM genes upon TGF-β stimulation, with a stronger effect size and higher statistical significance in female cells (mean logFC=1.6; mean *P*_adjusted_<0.001) compared with male cells (mean logFC=1.0; mean *P*_adjusted_=0.004; Figure S18C), although without significant sex interaction. Furthermore, 9 female fibrous genes were upregulated upon TGF-β stimulation (*P*_adjusted_<0.05; Figure 5G; Figure S18D), of which 4 genes showed SMAD3-dependent inhibition. The remaining genes may reflect alternative cellular mechanisms in female fibrous plaques, such as those involving ECs (Figure 4).

## Discussion

In this study, we show that women and men presenting with late-stage fibrous atherosclerotic plaques exhibit distinct risk factor profiles characterized by a high prevalence of smoking and diabetes, respectively. Our multiomic analysis shows that fibrous plaques from women are characterized by EndMT and SMC-driven ECM remodeling, whereas fibrous plaques from men presented a diabetes-related enrichment for inflammation driven by macrophages. We located the expression of the female-biased genes to the fibrous cap using spatially resolved transcriptomics. Furthermore, we experimentally confirmed that female candidate genes are upregulated in human plaque ECs upon exposure to TGF-β, suggesting that the molecular drivers of fibrous plaques may be sex-dependent.

Women who smoke have a 25% increased risk of developing coronary artery disease compared with men.^12^ Previous studies have demonstrated that smoking is associated with plaque erosion in women, which might explain the increased risk of acute coronary syndromes.^1^ In this study, we found that women with fibrous plaques are more likely to smoke compared with men with fibrous plaques. While sex differences in the prevalence of plaque erosion and fibrous plaques are especially pronounced at younger ages,^1,37^ women in our cohort (at an average age of 68 years) also have a higher prevalence of fibrous plaques compared with men, as consistently reported over the years.^8,38^ Men with fibrous plaques, on the other hand, are more likely to have diabetes, a risk factor known to be linked to the accumulation of macrophages and lipids.^39^

Our multiomics study demonstrates that patients presenting with late-stage fibrous atherosclerotic plaques, despite being histologically similar, present with distinct biological processes when stratified by sex. In-depth analyses of fibrous plaques at protein, gene, and DNA methylation levels revealed that female fibrous plaques are characterized by TGF-β, EndMT, and SMC-driven ECM remodeling. Similarly, a recent study from our group investigating the effect of smoking on the plaque transcriptome revealed a sex-dependent upregulation of SMC gene *CRLF1* in women, which is involved in ECM remodeling and exhibits the highest expression in fibrous-like plaque types.^40^ Spatial transcriptomics located the expression of the female fibrous genes primarily within the fibrous cap and media, which supports the hypothesis that fibrous plaques may be prone to endothelial dysfunction and erosion on the luminal side of the plaque. Spatial transcriptomics further revealed that the male-biased fibrous genes are expressed in regions of the plaque enriched with CD68^+^ staining, particularly within the intima and near the plaque core, suggesting that the expression of these genes is likely driven by macrophages. This aligns with previously identified sex differences in SMCs, ECs, and macrophages at the molecular level,^14,15^ as well as sex disparities in plaque proteomics,^41^ indicating that detailed analyses point toward diverging pathological mechanisms between the sexes even without clear histological differences.

These results also align with the distribution of molecular-based plaque phenotypes within our study population, where atheromatous-like phenotypes (lipomatous and fibroinflammatory) were more prevalent in fibrous plaques from men than women. It has been previously described that intrinsic differences in molecular mechanisms might influence the choice of drug treatments, particularly based on the molecular profiling for tumor subtypes in cancer.^42^ Similarly, the differences in mechanisms found in this study may have consequences for the domain of applications in emerging antifibrotic or anti-inflammatory therapies,^43,44^ which may vary in efficacy by sex and plaque phenotype.

EndMT and TGF-β pathways in ECs have been previously linked to atherosclerotic processes.^16,17,24^ Several analyses in this study highlight these pathways as key mechanisms involved in end-stage fibrous plaques in women. scRNAseq and methylation deconvolution further revealed a more prominent role for ECs and SMCs in fibrous plaques in women compared with men. Notably, recently published EndMT lineage markers^32^ were enriched within the female-biased fibrous genes identified in this study. In addition, our in vitro data using human plaque-derived ECs demonstrated that the female candidate genes are upregulated upon stimulation with TGF-β (and TNF-α). TGF-β stimulation was associated with a downward trend in endothelial marker expression and an upward trend in mesenchymal marker expression, resulting in the transition of ECs toward a mesenchymal phenotype. Importantly, inhibition of SMAD3 with SIS3 partially attenuated these transcriptional changes, suggesting that EndMT induction via TGF-β in plaque ECs is at least partly SMAD3-dependent. While we cannot establish direct causality through these in vitro experiments, we propose that TGB-β stimulation may induce EndMT, which plays a key role in the formation of fibrous plaques in women.

As ECs lose integrity and detach during EndMT, we hypothesize that this leads to plaque erosion.^45^ In both EndMT and plaque erosion, endothelial disruption is triggered by disturbed flow, endothelial shear stress, oxidative stress, and inflammatory stimuli.^5,17^ Although this hypothesis has been postulated before,^46^ future studies should be directed toward understanding whether EndMT is indeed causal for a disrupted EC layer and plaque erosion.

In addition to EndMT, our in vitro experiments with primary VSMCs demonstrate that TGF-β strongly induces expression of ECM remodeling pathways in a partly SMAD3-dependent manner. These findings complement our EC data and indicate that sex-based differences in fibrous lesions are mediated, at least in part, through TGF-β–SMAD3 signaling in both ECs and VSMCs.

Using our plaque epigenetics data, we provide circumstantial evidence that the average methylation of the female candidate gene promoters is significantly lower, specifically in fibrous plaques from women who smoke. These findings align with previous work on plaque epigenetics, underscoring how smoking can significantly impact genome-wide DNA methylation^13^ and gene expression,^40^ and influence epigenetic regulation in atherosclerotic disease. Furthermore, it has previously been demonstrated that the expression of EndMT genes is mediated through promoter hypomethylation upon TGF-β stimulation, a potent inducer of EndMT.^47,48^ Despite this, the exact relationship between risk factors, epigenetics, and atherosclerotic pathways requires further mechanistic research.

Our findings from patient-derived plaques reveal significant sex differences in atherosclerotic plaques, despite having similar histological composition. While we previously hypothesized that sex differences in atherosclerotic plaques were limited to variations in plaque composition, we now demonstrate that conducting sex-stratified analyses at the cellular and molecular levels is crucial even when the pathology appears similar between men and women. This highlights the importance of conducting such analyses and calls for the redesign of methods, such as SMC lineage tracing in mice. While these methods are now used in both male and female mice, they were previously limited to males due to the insertion of transgenes on the Y chromosome.^49^

This study has limitations. First, the histological plaque sections were scored using semiquantitative methods, which offer lower resolution compared with the molecular analyses of protein, gene, and DNA methylation. However, these histopathologic measures are widely used for plaque phenotyping and show strong replication and reliability.^38,50–52^ In addition, the classification of plaques into fibrous and atheromatous phenotypes was primarily based on the presence and size of the lipid core. To incorporate other histopathologic characteristics, we used the plaque vulnerability index.^15^ Notably, this index revealed clear differences between fibrous and atheromatous plaques yet showed no sex differences within these groups, thereby supporting our classification method. Another limitation is the low number of women in our study, decreasing the statistical power of our omics analyses. Therefore, we performed enrichment analyses using nominally significant differentially expressed genes and proteins. This may have introduced false positives, yet the pathways identified showed consistent signals across multiple omics layers.

To mitigate the impact of the sex imbalance in our cohort, we conducted propensity-matched analyses for the transcriptomics and proteomics data sets, creating cohorts with equal numbers of women and men. Matching was based on clinical presentation and classical atherosclerotic risk factors such as age, hypertension, diabetes, and smoking. These analyses revealed similar results between original and matched cohorts with strong correlations in effect sizes for all genes and proteins. While this matching strategy supports our conclusions, the limited number of females in our cohort significantly reduced our power, and we would like to emphasize the need for more inclusive studies to further support well-powered sex-stratified analyses.

We also acknowledge that we analyzed end-stage plaques, and while we identify ECM remodeling and EndMT as predominant processes in fibrous plaques in women, it remains unclear at which stage of plaque development these processes are important. This uncertainty is particularly relevant because women are less represented in the asymptomatic population and may present at a more advanced disease stage. In women, increased ECM remodeling and EndMT within the fibrous cap may make plaques more vulnerable to erosion, contributing to a higher risk of cardiovascular events. Targeting these processes could provide a therapeutic strategy to stabilize the fibrous cap and prevent acute events of plaque erosion more common in women.

In conclusion, women and men with end-stage fibrous atherosclerotic plaques reveal distinct clinical and molecular profiles. Female fibrous plaques are characterized by SMC-driven ECM remodeling and TGF-β pathways in ECs, which were located to the fibrous cap using spatial transcriptomics. Meanwhile, male fibrous plaques linked to macrophage-driven inflammation are located proximal to the core, dependent on diabetes. These mechanisms could be key to understanding plaque erosion at the molecular level and offer novel targets for atherosclerosis therapies, as they account for both sex and plaque phenotype.

## ARTICLE INFORMATION

### Acknowledgments

The authors would like to acknowledge all the participants of the Athero-Express Biobank for agreeing to be part of the study.

### Sources of Funding

This work has been supported by the ERC Consolidator Grant UCARE 866478, the Leducq Foundation Transatlantic Network of Excellence AtheroGEN and PlaqOmics, and the Chan Zuckerberg Initiative MetaPlaq. S.W. van der Laan is funded through EU H2020 TO_AITION (grant 848146), EU HORIZON NextGen (grant 101136962), EU HORIZON MIRACLE (grant 101115381), and the Health≈Holland PPP Allowance, Getting the Perfect Image.

### Disclosures

S.W. van der Laan received Roche funding for unrelated work. Roche had no part in this study, neither in the conception, design, and execution of this study, nor in the preparation and contents of this manuscript. The other authors report no conflicts.

### Supplemental Material

Supplemental Materials and Methods

Tables S1–S29

Figures S1–S18

Major Resources Table

References 53–69

## Supplementary Material


