# Supplementary material for: Atherosclerotic Fibrous Plaques in Women Present ECM Remodeling Linked to TGF-β
Source: Circ Res. 2026 Feb 5;138(5):e327624. doi: 10.1161/CIRCRESAHA.125.327624 (PMC12947913; doi:10.1161/CIRCRESAHA.125.327624)
Supplement: Supplementary file 1 [file res-138-e327624-s001.pdf]

Supplemental Material

Atherosclerotic fibrous plaques in women present ECM remodeling linked to TGF-β

Short title: **Atherosclerotic fibrous plaques in women**

Tim R. Sakkers [1], Eloi Mili [1], Hanna Winter [2, 3], Daniek Kapteijn [1], R. Noah Perry [4,5], Nicolas Barbera [5], Kelsey Watts [5], Inês R. Dias [1], Denitsa Meteva [6], Marian Wesseling [7], Barend M. Mol [8], Gert J. de Borst [8], Dominique P.V. de Kleijn [8], Sander W. van der Laan [7], Mete Civelek [9,10,11,12], Stephen J. White [13], Lars Maegdefessel [2, 3], Manuel Mayr [14, 15], Gerard Pasterkamp [7], Michal Mokry [1, 7], Ernest Diez Benavente* [1], Hester M. den Ruijter* [1]

Affiliations:

[1] Laboratory of Experimental Cardiology, University Medical Center Utrecht, Utrecht University, The Netherlands.

[2] Institute of Molecular Vascular Medicine, TUM Klinikum, Technical University Munich, Germany.

[3] German Center for Cardiovascular Research, partner site Munich Heart Alliance, Berlin, Germany.

[4] Department of Biomedical Engineering, University of Virginia, Charlottesville, VA.

[5] Department of Genome Sciences, University of Virginia, Charlottesville, VA.

[6] Charité-Universitätsmedizin Berlin, Corporate member of Freie Universität Berlin and Humboldt-Universität zu Berlin, Germany.

[7] Central Diagnostic Laboratory, University Medical Center Utrecht, Utrecht University, The Netherlands.

[8] Department of Vascular Surgery, University Medical Centre Utrecht, Utrecht University, The Netherlands.

[9] Department of Anesthesiology and Perioperative Medicine, University of California, Los Angeles, CA.

[10] Department of Human Genetics, University of California, Los Angeles, CA.

[11] Division of Cardiology, Department of Medicine, University of California, Los Angeles, CA.

[12] Institute for Precision Health, David Geffen School of Medicine, University of California, Los Angeles, CA.

[13] Biosciences Institute, Faculty of Medical Sciences, Newcastle University, United Kingdom.

[14] National Heart and Lung Institute, Imperial College London, London, UK.

[15] Department of Internal Medicine II, Division of Cardiology, Medical University of Vienna, Austria.

* These authors contributed equally

Expanded Materials & Methods

1 Study population

The Athero-Express Biobank (AE) is an ongoing longitudinal biobank since 2002 aimed at investigating atherosclerotic plaques in patients undergoing arterial endarterectomy. Clinical data is obtained through baseline blood withdrawal and extensive questionnaires filled in by participants that are verified against medical records. We analyzed patients who underwent carotid endarterectomy (CEA) and had available protein, gene, and DNA methylation plaque data (Supplemental Fig. 1A). Every single plaque included in this study originates from one patient, ensuring that no sequential bilateral procedures are included. The methods for plaque EC isolations are described below.

2 Atherosclerotic plaque histology

The atherosclerotic plaque was immediately processed following surgical removal. As previously documented [15,38,50,53], (immune-)histochemical staining was routinely performed on the segment with the most significant plaque burden (culprit lesion) for identification of macrophages (CD68), calcification (hematoxylin-eosin), SMCs (alpha actin), collagen (picro sirius red), plaque hemorrhage (hematoxylin-eosin, Elastin von Gieson staining), vessel density (CD34), and fat (picrosirius red, hematoxylin-eosin). Plaques underwent a semiquantitative assessment, and their classification into fibrous, fibroatheromatous, or atheromatous plaques was based on lipid core presence and size, which was carried out in accordance with previously described scoring protocol [50]. The consistency between and among observers was examined previously and showed good concordance (κ=0.6–0.9) [52]. The culprit lesion was used for histology, while the plaque segments closest to the culprit lesion were used for sequencing. The plaque vulnerability index is a combined score (ranging from 0 to 4) of individual histological features (SMC content, collagen content, lipid content, and macrophage content) [15]. Plaque erosion is defined as the degradation or loss of the endothelial layer covering an atherosclerotic plaque, which can lead to the exposure of the underlying tissue and result in acute thrombus formation. Given its association with fibrous plaques, which are more prone to erosion, these plaques are the primary focus of our study [4–7].

3 Propensity score matching

To account for the potential effect of sex imbalances, we performed propensity score matching analyses to create cohorts with equal numbers of men and women. The first matching analysis was based on key atherosclerotic risk factors, including age, smoking status, diabetes, and hypertension. The second analysis was based on clinical presentation, categorized as asymptomatic, ocular symptoms, transient ischemic attack (TIA), or stroke. Both analyses were conducted with the matchit package in R, using the optimal matching method and logistic regression to estimate propensity scores. Covariate balance was assessed using standardized mean differences, visualized with Love plots with a balance threshold of <0.1. Transcriptomics and proteomics data were subsequently analysed in both the original and matched cohorts, and effect sizes were compared to evaluate consistency. To assess whether statin use affected our findings, we performed transcriptomic and scRNAseq analyses separately for patients with and without statin prescriptions.

4 Bulk RNA sequencing of atherosclerotic plaques

Out of all carotid plaques (n=1,889), 191 plaques (female: 61, male: 130) were used for RNA-sequencing to study sex differences in fibrous plaques at the gene expression level (Supplemental Table 4). For the atheromatous plaque comparison, we selected 187 plaques (female: 29, male: 158). Isolation, library preparation, sequencing, and preprocessing of plaque RNA were previously documented [15,21]. In short, total RNA was extracted from plaque segments using ceramic beads and a tissue homogenizer with use of TriPure. Library preparation for RNA sequencing involved reverse transcription, incorporating a primer mix

and unique barcodes for each sample, facilitating pooling of cDNA samples. The cDNA underwent in vitro transcription, primer removal, fragmentation, and purification. RNA quality was assessed using a Bioanalyzer, and cDNA libraries were constructed for sequencing on the Illumina NextSeq500 platform with paired-end reads. Post-sequencing, data were processed for mapping and analysis, employing the Burrows-Wheel Aligner and custom scripts for generating count matrices from sequence reads.
Further analyses were performed using R-3.6.2 and its IDE Rstudio version 1.2 and later. Genes were annotated with Ensembl ID's and differential gene expression analysis was performed using the DESeq2 R-package [54]. Including sex chromosomes in our analysis revealed them as the most differentially expressed, accounting for 88% of the top 100 differentially expressed genes (Supplemental Fig. 2A). These genes were removed from subsequent analyses. We selected genes that were differentially expressed ($p<0.05$, logFC>0.3, baseMean>10) between female and male fibrous plaques and female and male atheromatous plaques (Supplemental Fig. 2B). Differentially expressed genes were enriched using clusterProfiler [55].

5 Untargeted LC-MS proteomics in atherosclerotic plaques
Untargeted proteomics was conducted on 125 plaques to study sex differences in fibrous (female: 19, male: 46) and atheromatous (female: 14, male: 46) plaques at the protein level (Supplemental Table 12). More details regarding these methods can be found below.
Data analysis involved a database search against the human UniProtKB/Swiss-Prot database with specific modifications and enzyme settings. Protein identification yielded 2,148 proteins, with ECM and related proteins categorized using Matrisome DB and further in-house selection. Protein abundances were filtered, normalized, and scaled, with missing values imputed using the KNN-Impute method, resulting in a final dataset of 1,499 proteins for further analysis. Differential abundance analysis was performed using Limma [56]**.** We selected proteins that were differentially abundant between female and male fibrous plaques ($p<0.05$, logFC>0.3, AveExpr>4). Differentially abundant proteins were enriched using clusterProfiler [55].

Protein extraction
Tissue sections were diced and weighed, and approximately 40-80 mg of tissue was taken for 2-step protein extraction, similar as previously described but skipped the SDS extraction step [57]. Briefly, samples were incubated in a NaCl extraction buffer (0.5 M NaCl, 25 mM EDTA, 10 mM Tris pH 7.5, plus protease inhibitors) with slow agitation for 2 h. The NaCl fraction (supernatant) was transferred to a new tube and stored at -80°C for later use. Subsequently, samples were incubated in a guanidine hydrochloride buffer (4 M GuHCl, 50 mM sodium acetate pH 5.8, 25mM EDTA and protease inhibitors) for 48 h to solubilize cellular proteins and long-lived ECM proteins. Protein concentrations of GuHCl extracts were quantified using a Pierce BCA protein assay kit according to the manufacturer's instructions (Pierce BCA Protein Assay Kit, 23225, Thermo Scientific).

Deglycosylation
A two-step deglycosylation protocol was employed for GuHCl extracts. First, 20ug of proteins from each sample were precipitated using 10x volume of ethanol overnight. Next, samples were centrifuged at 4°C, 16,000xg for 30min. Afterwards the supernatant was discarded, and the protein pellets were dried using a SpeedVac (Thermo Scientific, Savant SPD131DDA). The protein pellets were resuspended in deglycosylation buffer (150 mM NaCl, 50 mM sodium acetate, 10 mM EDTA, pH 6.8, plus protease and phosphatase inhibitors) containing the following deglycosylation enzymes: Endo-α-N-acetylgalactosaminidase, α2-3,6,8,9-Neuraminidase, β-1,4-Galactosidase, β-N-Acetylglucosaminidase (all Merck-Millipore Glycoprotein Deglycosylation Kit, 362280), Chondroitinase ABC (Sigma-Aldrich, C3667), Heparinase II (Sigma-Aldrich, H6512), and Endo-β1,4-galactosidase (Sigma-Aldrich, G6920). Samples were incubated at 25 °C for 2 h, followed by 37 °C for 24 h. Second, samples were

dried using a SpeedVac, reconstituted in O18-labeled water (Taiyo Nippon Sanso, F03-0027) containing PNGase F (Merck-Millipore, 362280), and incubated at 37 °C for 48 h.

In-solution digestion

Proteins were denatured using 6 M urea and 2 M thiourea and reduced with 10 mM DTT at 37 °C for 1 h. The samples were then cooled to room temperature before being alkylated using 50 mM iodoacetamide followed by incubation in the dark for 45 min. Pre-chilled (-20 °C) acetone (10x volume) was used to precipitate the samples overnight at -20 °C. Samples were centrifuged at 14,000 x g for 40 min at 4 °C and the supernatant was subsequently discarded. Protein pellets were dried using a SpeedVac, resuspended in 0.1 M triethylammonium bicarbonate (TEAB) buffer, pH 8.2, containing mass spectrometry grade trypsin/LysC (Promega) (1:50 trypsin: protein), and digested overnight at 37 °C. The digestion was stopped by acidification of the samples with a final concentration of 1% trifluoroacetic acid (TFA). Peptide samples were then purified using C18 cartridges on a Bravo AssayMAP robotic system (Agilent) according to the manufacturer's instructions. The dried peptide was reconstituted with 2% acetonitrile (ACN), 0.05% TFA in water.

LC-MS/MS

Peptides was separated by a nanoflow LC system (Dionex UltiMate 3000 RSLC nano). Samples were injected onto a nano-trap column (Acclaim PepMap100 C18 Trap, inner diameter 300 µm x length 5 mm, particle size 5 µm, pore size 100 Å), at a flow rate of 25 µL/min for 3 min, using 0.1% formic acid (FA) in water. The following nano-LC gradient was then run at 0.25 µL/min to separate the peptides: 0−1 min, 1% B; 1−6 min, 1–6% B; 6–40 min, 6–18% B; 40-70 min, 18-35% B; 70–80 min, 35–45% B; 80–81 min, 45–99% B; 81–89.8 min, 99% B; 89.8–90 min, 99–1% B; 90–120 min, 1% B; where A = 0.1% FA in water, B = 80% ACN, 0.1% FA in water. The nano column (EASY-Spray PepMap RSLC C18, 75 µm x 500 mm, 2 µm, 100 Å), set at 45 °C, was connected to an EASY-Spray ion source (Thermo Scientific). Spectra were collected from an Orbitrap mass analyzer (Q Exactive HF, Thermo Scientific) using full MS mode over the mass-to-charge (m/z) range 350–1600 with resolution of 60,000 at 200 m/z. Data-dependent MS2 scan was performed using Top15 method with HCD activation and Orbitrap detection with resolution of 15,000 in each full MS scan with dynamic exclusion enabled. Thermo Scientific Proteome Discoverer software (version 2.4.1.15) was used to search raw data files against the human database, (UniProtKB/Swiss-Prot version 2021_01, 20,396 protein entries) using Mascot (version 2.6.0, Matrix Science). The mass tolerance was set at 10 ppm for precursor ions and 20 milli mass unit (mmu) for fragment ions. Trypsin was used as the digesting enzyme with up to two missed cleavages being allowed. Carbamidomethylation of cysteine was chosen as a static modification and oxidation of methionine, proline and lysine, and deglycosylation with presence of O18-water on asparagine were chosen as a dynamic modification. Protein identification FDR confidence was set to High and a minimum number of peptides per protein was 2. Only Master proteins were shown. Precursor peak area was used for quantification and normalized to total peptide peak area of each sample.

6 Spatial transcriptomics of human carotid plaques

Untargeted human carotid plaque samples for spatial transcriptomic analysis were derived from CEA and received from the Munich Vascular Biobank [30]. The carotids were fixed for 24 hours in 4% paraformaldehyde at room temperature, decalcified (EDTA-basis), paraffin embedded and cut into 5µm thick sections which were then mounted on Xenium slides (10X Genomics, Pleasanton, USA). Then, the manufacturer's Xenium protocol was applied (10X Genomics, Pleasanton, USA). The resulting images were analysed using the provided software Xenium Explorer 3 (version 3.0.0.; 10X Genomics). For confirmatory staining, Hematoxylin and Eosin and CD68 immunohistochemistry was performed as previously described [58]. From this biobank, we selected one female plaque with an intact fibrous cap and one male plaque with an intact fibrous cap.

7 Single-cell RNA sequencing of atherosclerotic plaques

Single-cell RNA sequencing (scRNAseq) was performed on 46 plaques (female: 20, male: 26) to study cell-type-specific expression patterns of differentially expressed genes (Supplemental Table 22). The process of preparing, sorting, and sequencing cells from plaques was described in detail previously [59,60]. scRNAseq data was processed in R-3.6.2 using the Seurat-3.2.2 R-package [61]. Mitochondrial genes and doublets were filtered out from the data, setting thresholds for unique and total reads per cell. Batch effects were corrected using SCTransform. Clustering was performed with 20 principal components, validated through a JackStraw analysis to ensure significant feature distribution. A clustering resolution of 0.8 was chosen to reflect meaningful biological groupings without overfitting. Sensitivity analysis showed minimal impact of clustering parameters on cell-type identification. Optimal clustering parameters were finalized after multiple iterations. Cell populations were annotated using differential gene expression analysis and automated cell type classification was performed with SingleR (version 1.2.4) comparing cluster profiles to the BLUEPRINT reference dataset. ECs were identified by high expression of *PECAM1* and *VWF*, while SMCs were classified by expression of *ACTA2* and *TAGLN*. Subpopulations of SMCs and ECs were identified by further clustering using 10 principal components at a higher resolution, and distinct identities were assigned based on differential gene expression analyzed via enrichR-3.0 [62]. The module score for differentially expressed genes was calculated using the addModuleScore function in Seurat [61], providing an expression proxy for gene sets. Genes that were not represented in the scRNAseq were removed from this calculation.

8 DNA methylation on atherosclerotic plaques

DNA methylation data was obtained from 102 fibrous plaques (female: 38, male: 64) to explore cell-type deconvolution and the potential regulation of differentially expressed genes between the sexes (Supplemental Table 23). For the atheromatous plaque deconvolution, we selected 117 plaques (female: 22, male: 95). DNA isolation, preparation, and methylation conversion were previously described [13,63]. In short, the purity and concentration of DNA were evaluated using the Nanodrop 1000 system (Thermo Scientific). DNA samples were standardized to a concentration of 600 ng/ml, distributed randomly across 96-well plates, and then subjected to bisulfite conversion through a cycling protocol using the EZ-96 DNA Methylation Kit (Zymo Research). Subsequently, DNA methylation was measured on the Infinium HumanMethylation450 Beadchip Array at the Human Genotyping Facility of the Erasmus Medical Center. Quality control, normalization, and filtering of the methylation data were performed following an established cross-package Bioconductor workflow, using the Limma and Minfi packages [64]. CpG filtering was performed on probes located on sex chromosomes, failed in one or more samples, contained SNPs or mapped to multiple places in the genome.

9 Cell-type deconvolution

To build our human methylation atlas, we adopted a previously published approach[65], using methylation data from 39 cell types sorted from 205 healthy tissue samples. In summary, this process involved selecting the top 100 most specific hyper-and hypomethylated CpGs for a selection of cell types (n=8) expected to be present in atherosclerotic plaques: ECs, SMCs, erythrocyte progenitors, monocytes/macrophages, NK cells, T cells, and B cells. We calculated the proportion of each CpG relative to the overall methylation pattern across these cell types. For each cell type, we then selected the top 100 hypermethylated CpGs with the highest contributions, considering their relative methylation levels. To identify the top 100 hypomethylated CpGs, we used the reversed methylation values (1-beta value). To determine the relative contribution of each cell type (%) to the atherosclerotic plaque, we performed deconvolution using non-negative least squares from the nnls package in R. We used the DNA methylation atlas from healthy cells for deconvolution after showing that there is high degree of methylation similarity between healthy and plaque-derived SMCs

(Supplemental Fig. 11). This demonstrates that DNA methylation from healthy cells can be used for deconvolution studies, including those involving complex disease samples, aligning with findings from previous studies [65].
For bulk RNA-seq deconvolution, we used Scaden (v1.1.2), a deep learning-based deconvolution method, to estimate cell type proportions from bulk RNA-seq data by training on simulated mixtures generated from single-cell RNA-seq references, as previously described [66].

10 Endothelial cell isolation and expansion for *in vitro* experiments
Plaque ECs were isolated from fresh atherosclerotic plaque tissues obtained from patients undergoing carotid endarterectomies (7 female, 11 male donors, Supplemental Table 24). Fresh plaques were collected in Hanks Balanced Salt Solution within 15 minutes of operative resection. To detach ECs, the lumen of the plaque tissues was treated with an enzyme mixture containing 1 mg/mL animal-free Collagenase/Dispase Blend II (Merck, REF#SCR140) and incubated for 5 minutes at 37°C in a 5% CO_2 incubator. After enzymatic digestion, the lumen was swabbed, and the swab was washed multiple times with complete medium consisting of basal EC growth medium (EBM-MV, phenol red-free; Promocell, REF#C-22225) supplemented with an EBM cell growth MV Kit (Promocell, REF#C-39220) and 1% Penicillin/Streptomycin (Fisher Scientific, REF#15-140-122). During the first week of culture, the antimicrobial agent Primocin (Invivogen, REF#ant-pm-2) was added to the medium. Thereafter, cells were cultured without Primocin. The ECs were plated in a 0.1% gelatin-coated 12-well plate and incubated at 37°C in a 5% CO_2 incubator. The medium was refreshed every 2–3 days. Colonies of ECs typically appeared between 7 to15 after the procedure . Upon reaching 80–90% confluency, colonies were further cultured and sequentially transferred from 12-well plates to 6-well plates and eventually into T75 flasks. Cell counts were performed using the TC20 automated cell counter (Bio-Rad Laboratories, Hercules, CA). A total of 130,000 cells per well were seeded in a 12-well plate and incubated at 37°C in a 5% CO_2 incubator for 24 hours to form a confluent monolayer. Cells were monitored for confluence (95–100%) before initiating TGF-β stimulation. HCAECs were cultured until 80% confluency and 324 seeded in a 6-well plate with a concentration of 260.000 cells per well. After a 24-hour 325 incubation at 37°C in 5% CO2, cells were used for TGF-β stimulations, as described below.

11 Plaque endothelial cell immunofluorescence staining
ECs were fixed using 4% paraformaldehyde (PFA) for 15–20 minutes at room temperature and subsequently washed three times with phosphate-buffered saline (PBS). Fixed cells were permeabilized with 0.1% Triton X-100 in PBS for 10 minutes, followed by three additional PBS washes. To block nonspecific binding, slides were incubated with 10% normal goat serum in PBS for 60 minutes. Cells were incubated overnight at 4°C with primary antibodies diluted in 1% PBS Albumin. Cells were stained overnight with von Willebrand factor (CAT#A0082; DAKO, 1:300 dilution) and Ve-Cadherin (CAT# SC-9989; Santa Cruz Technologies, 1:400 dilution)The next day, cells were washed three times with PBS and incubated for 60 min with goat anti-mouse AF488 (FITC, Fisher Scientific, CAT#A11001) and goat anti Rabbit AF555 (CAT#A21428, Fisher Scientific). Following incubation, cells were washed three times with PBS. To counterstain nuclei, slides were incubated with Hoechst (1:10,000 in PBS) for 2–3 minutes. A final PBS wash was performed, and slides were mounted with Fluoromount and covered with coverslips. Slides were either analyzed immediately or stored in the dark at 4°C for short-term use or at -20°C for long-term storage.

12 Flow cytometry characterization of plaque endothelial cells
Plaque ECs were dissociated at passage 3 to 6 and were dissolved with 50 µl FACs buffer, which consisted of PBS (REF#10010056; Gibco) supplemented with 5% FBS (REF#35-079-CV; Corning) and 0.2% EDTA (REF#101448118; ASC Reagent). The cells were stained with CD34 (REF#303121; Biolegend), PECAM1 (REF#564105; BD Pharmingen), Ve-Cadherin

(REF#348515; Biolegend), CD14 (REF#301839, Biolegend), CD45 (REF#348516, Biolegend), CD41 (REF#303726; Biolegend). All antibodies were diluted 1:40 into a final volume of 100 µl PBS and incubated for 30 min at 4°C. Thereafter, cells were washed with 2 ml FACs buffer and stained with live/dead discriminator Zombie NIR (REF#423105, Biolegend) diluted 1:1000 in PBS. Subsequently, cells were resuspended in 250 µl FACs buffer and measured using CytoFLEX (Beckman Coulter) and analyzed using Kaluza Analysis Version 2.1 (Beckman Coulter). Plaque myofibroblasts[67], human coronary artery endothelial cells (HCAEC, Lonza; Female; REF#CC-2585), human umbilical vein endothelial cells (HUVECs, TCBio 18-234), peripheral blood mononuclear cells (PBMCs, Mini Donor Service, University Medical Center Utrecht; code: F1P150) were used as reference cells.

13 TGF-β (+TNFα) stimulation and SIS3 inhibition of endothelial cells

EC stimulation with TGF-β and TNFα has been widely documented and used as a model to study EndMT [68,69]. Plaque ECs were seeded in 12-well plates and cultured in complete EC medium until a confluent monolayer was obtained. Cells were then switched to starvation medium (endothelial basal medium, MV, phenol red-free; Promocell, REF#C-22215, 5% charcoal stripped FBS; ThermoFischer Scientific, REF#12676029) supplemented with 10 ng/ml TGF-β (Peprotech, REF#100-35) and/or 25 ng/ml TNFα (Miltenyi Biotec, REF#130-094-018). Duplicate wells were prepared for each condition, including a negative control with only starvation medium. Starvation medium containing the indicated stimuli was replaced after 48 and 96 hours after initial stimulation. EndMT progression was assessed at different timepoints by evaluating cell morphology, RNA expression and flow cytometry. To inhibit SMAD3-dependent TGF-β signaling, cells were pre-treated with SIS3 (Merck, REF#566405-1MG) in starvation medium prior to TGF-β stimulation and maintained under the same treatment schedule. RNA collected in 200µL TriPure (REF#11667165001; Roche) at 144 hours after the first stimulation. HCAECs cells were incubated at 37°C in 5% CO2 for 24 hours, after which they were treated with human TGF-β alone or in combination with TNFα (10 ng/mL each) in endothelial basal medium MV supplemented with 0.5% fetal bovine serum. The media and stimuli were refreshed after 48 hours. RNA was isolated at baseline (0 hours) and after 72 hours of stimulation, and cell morphology was monitored continuously via microscopy. Detailed protocols for staining, qPCR, and RNA analysis can be found elsewhere [32,63]. Genes were annotated using Ensembl IDs, and differential gene expression analysis was conducted using the DESeq2 R package [54].

14 Flow cytometry characterization of activated plaque endothelial cells

Activation markers of stimulated plaque ECs were measured using flow cytometry. After stimulation at 24 and 144 hours ECs were dissolved in 50µl FACs buffer. ECs were stained with ICAM (Biolegend, REF#322713), VCAM (Biolegend, REF#305815), P-selectin (Biolegend, REF#304941), E-selectin (Biolegend, REF#336015), and Tissue Factor (CD142, Biolegend, REF#365203). All antibodies were diluted 1:40 into a final volume of 100 µl PBS and incubated for 30 min at 4°C. Thereafter, cells were washed with 2 ml FACs buffer and stained with live/dead discriminator Zombie NIR (REF#423105, Biolegend). Subsequently, cells were resuspended in 250 µl FACs buffer and measured using CytoFLEX (Beckman Coulter) and analysed using Kaluza Analysis Version 2.1 (Beckman Coulter).

15 Monocyte adhesion assay

To investigate interactions between activated endothelial cells and monocytes, we employed a fluorescence-based adhesion assay in combination with the ibidi flow system. Female and male plaque ECs (Supplemental Table 24) were cultured to ~80% confluency. A total of 3 × 105 endothelial cells were seeded into µ-Slide I Luer slides (0.4 mm channel height; ibidi, REF#80176) and incubated for 2 hours at 37 °C, 5% CO_2 before connection to the ibidi pump system. The µ-slides were connected to an ibidi perfusion set (yellow/green, 50 cm, ID 1.6 mm; REF#10964), which was filled with 12 ml complete EC medium. Cells were cultured overnight under flow (5.8 mbar pressure, shear stress: 4.0 dyn/cm², flow rate: 3.04 ml/min,

shear rate: 400 s^{-1}, unidirectional: 20 s, oscillating: 0.5 s). The following day, complete medium was replaced with 12 ml starvation medium. Endothelial cells were either stimulated with 10 ng/ml TNFα for 5 hours at 37 °C, 5% CO_2 or left untreated (control). In parallel, 2 × 106 THP-1 monocytes were harvested and labeled with CellTrace CFSE Cell proliferation kit (ThermoFischer Scientific, REF#C34554). 1 × 106 THP-1 cells were introduced into each perfusion system and allowed to circulate for 2 hours under the same flow conditions. After perfusion, µ-slides were removed, rinsed with PBS, imaged, and subsequently fixed with 4% paraformaldehyde.

16 Quantitative PCR (qPCR)
Approximately 500ng of RNA was reverse transcribed using QScript cDNA synthesis Kits (REF#95047-100, QuantaBio). qPCR experiments were performed using Perfecta SYBR Green SuperMix (REF#95054-02K, QuantaBio), and Bio-Rad CFX Connect device. Threshold cycle values (Ct) were analyzed, using the 2−ΔΔCt method. Primers used are shown in Supplemental Table 24; HPRT1, YWHAZ and β-actin were used as household genes to correct for cDNA input.

17 Vascular smooth muscle cell isolation and stimulation
Vascular smooth muscle cells (VSMCs) were isolated from ascending aortic explants obtained from five male and five female donors [20]. Cells were seeded in 24-well plates and cultured in complete growth medium (Smooth Muscle Cell Basal Medium supplemented with Smooth Muscle Medium-2 SingleQuots Kit, Lonza) until they reached approximately 70% confluence. The medium was then replaced with serum-free medium for 24 hours to induce quiescence. After serum deprivation, each donor's VSMCs were cultured for an additional 24 hours in either serum-free medium alone (control), serum-free medium containing 20 ng/mL transforming growth factor-β1 (TGF-β1, R&D Systems), or serum-free medium containing 20 ng/mL TGF-β1 and 5 µM SIS3 (R&D Systems). Following treatment, total RNA was extracted from each well using a Qiagen RNA Extraction Kit, yielding a total of 18 RNA samples (six donors × three conditions).

18 Bulk RNA sequencing of vascular smooth muscle cells
RNA integrity was assessed on an Agilent TapeStation, and only samples with RNA Integrity Number (RIN) ≥ 9.0 were used for library preparation. Strand-specific RNA-seq libraries were prepared using the Illumina TruSeq Stranded mRNA kit according to the manufacturer's instructions. Poly(A)+ RNA was enriched from 1 µg of total RNA using oligo(dT) beads, fragmented to an average size of ~200 bp, and reverse-transcribed to generate cDNA. After second-strand synthesis incorporating dUTP for strand specificity, end repair, A-tailing, and adapter ligation were performed. Libraries were PCR-amplified for 10 cycles and purified with AMPure XP beads to remove adapter dimers. Library size distributions were checked on a Bioanalyzer High Sensitivity DNA chip before sequencing. Pooled libraries were sequenced on an Illumina NovaSeq 6000 platform using paired-end 150 bp reads (PE150), generating an average depth of 40–60 million read pairs per sample. PhiX control libraries were spiked in to monitor run quality. Raw FASTQ files underwent initial quality assessment with FastQC v0.11.9. Adapters and low-quality bases were trimmed with Trimmomatic v0.39, removing bases with Phred scores <20 and discarding reads shorter than 36 bp after trimming. Filtered reads were aligned to the GRCh38 (hg38) human reference genome using STAR v2.7 with a two-pass mapping strategy to improve splice junction detection. Only uniquely mapped read pairs were retained for downstream analysis. Alignment quality was further assessed using samtools v1.11 and Picard v2.23.9 to compute metrics such as mapping rate, insert size distribution, and ribosomal RNA contamination.Transcript abundance was quantified with RSEM v1.3, using the GENCODE v38 primary assembly annotation. RSEM's expectation–maximization algorithm probabilistically assigned multi-mapping reads and reported both expected counts and normalized values in transcripts per million (TPM). For gene-level analyses, expected counts were aggregated across transcripts.

Supplemental Figures

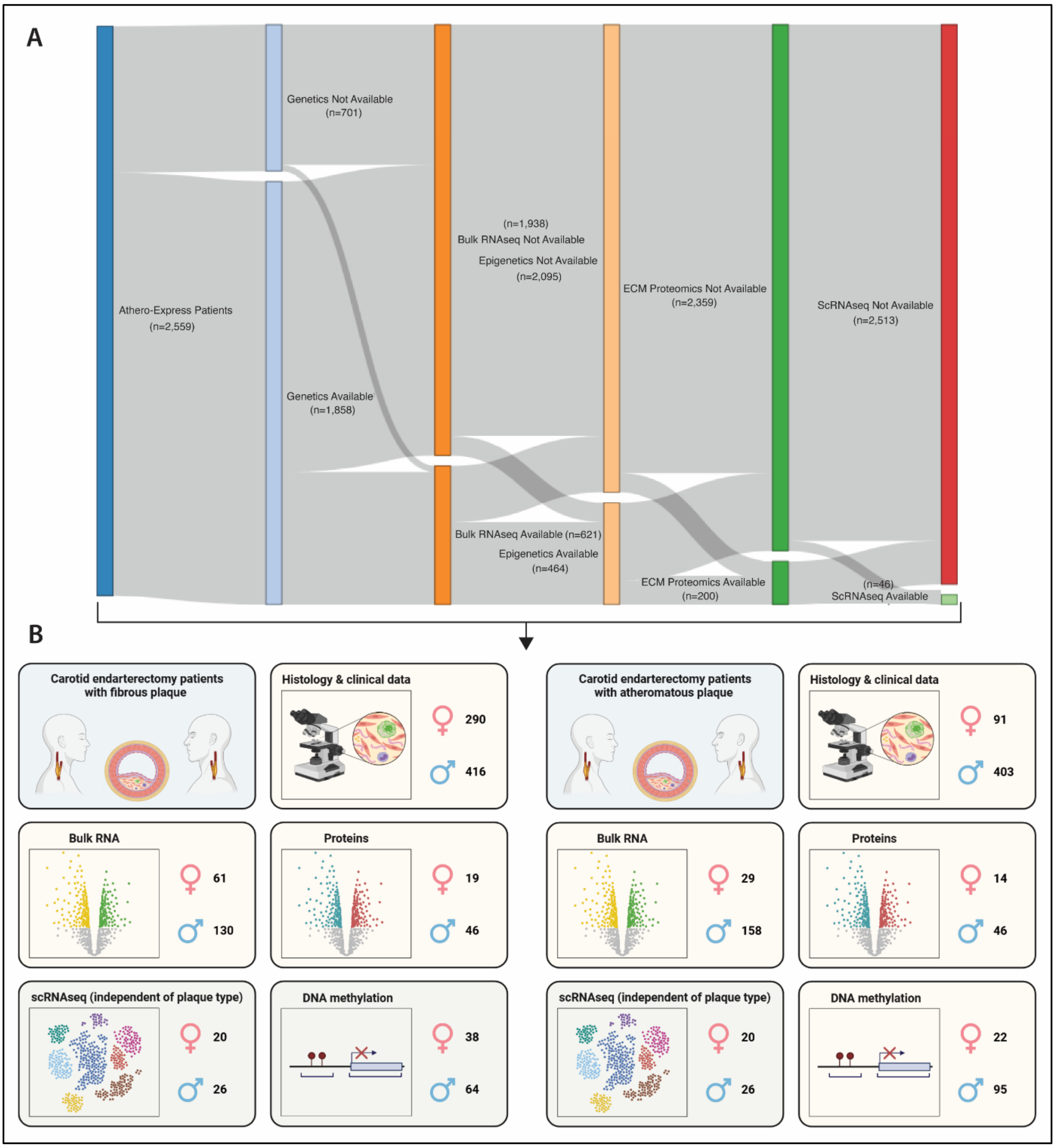

***Supplemental Figure 1: Overview of the available ómics data for the Athero-Express biobank**. Diagram illustrating the Athero-Express patient cohort and the overlap in available ‘omics datasets (**A**). Overview of the study’s patient population with fibrous and atheromatous plaques, including clinical, histology, bulk RNA, protein, scRNAseq and DNA methylation data (**B**).*

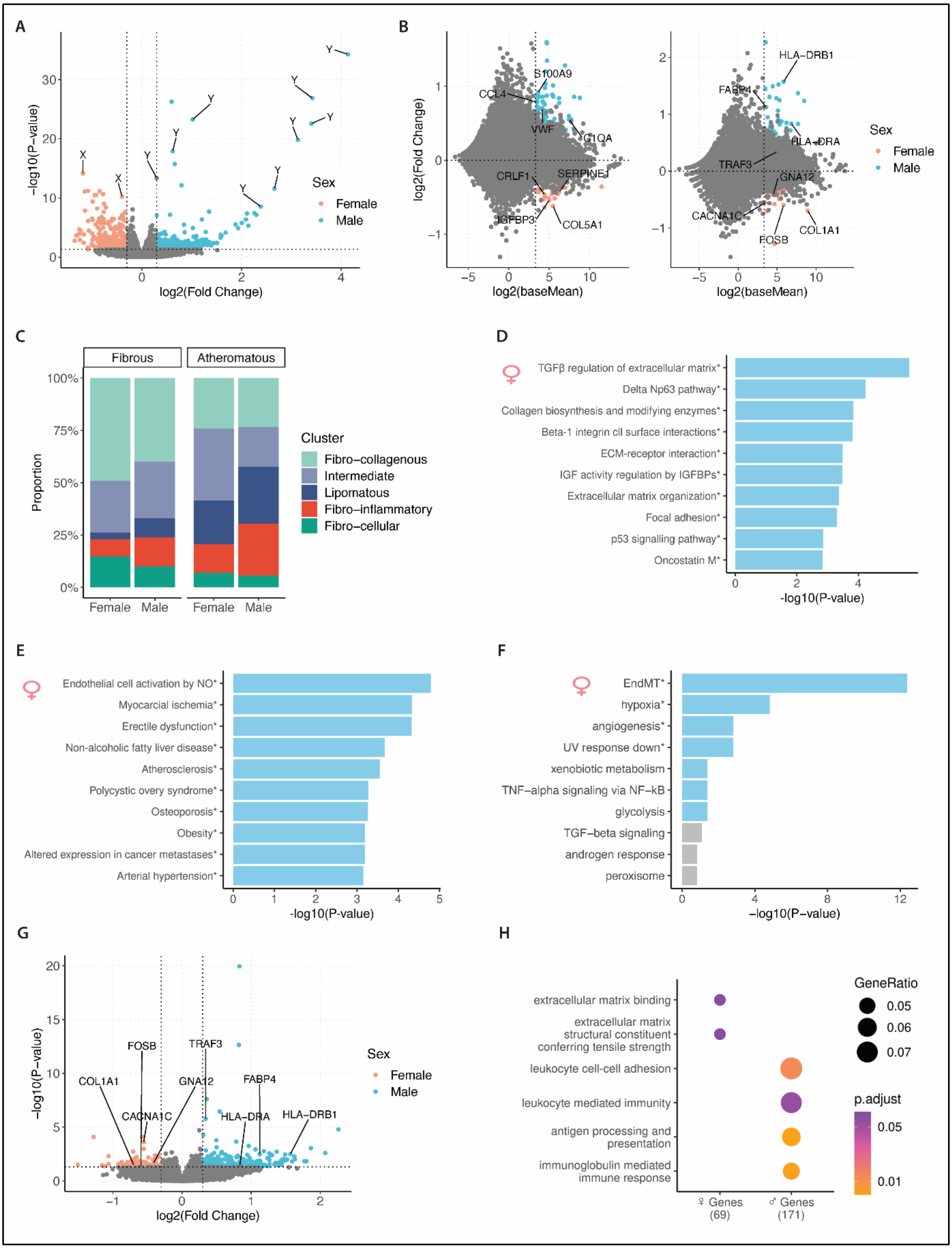

Supplemental Figure 2: Transcriptomic and proteomic sex-differences in fibrous and atheromatous plaques. *A volcano plot is shown for the differential gene expression analysis between fibrous plaques from female and male patients including genes from sex chromosomes (**A**). MA plot for the differential gene expression*

*analysis between female and male fibrous plaques and female and male atheromatous plaques (**B**). Assignment of atherosclerotic plaques to transcriptomic-based clusters based on gene expression patterns (**C**). Gene enrichment analysis for female-biased genes based on the BioPlanet database (**D**). Gene enrichment analysis for female-biased genes based on the Elsevier Pathway Collection database (**E**). Gene enrichment analysis for female-biased genes based on the MSigDB Hallmark database (**F**). A volcano plot is shown for the differential gene expression analysis between atheromatous plaques from female and male patients (**G**). GO enrichment analyses for female-biased (left panel **H**) and male-biased genes (right panel **H**). Statistical tests: DESeq2 Negative binomial GLM + Wald test (**A, B** & **G**), and Hypergeometric test (**D**-**F**), BH-FDR (**H**).*

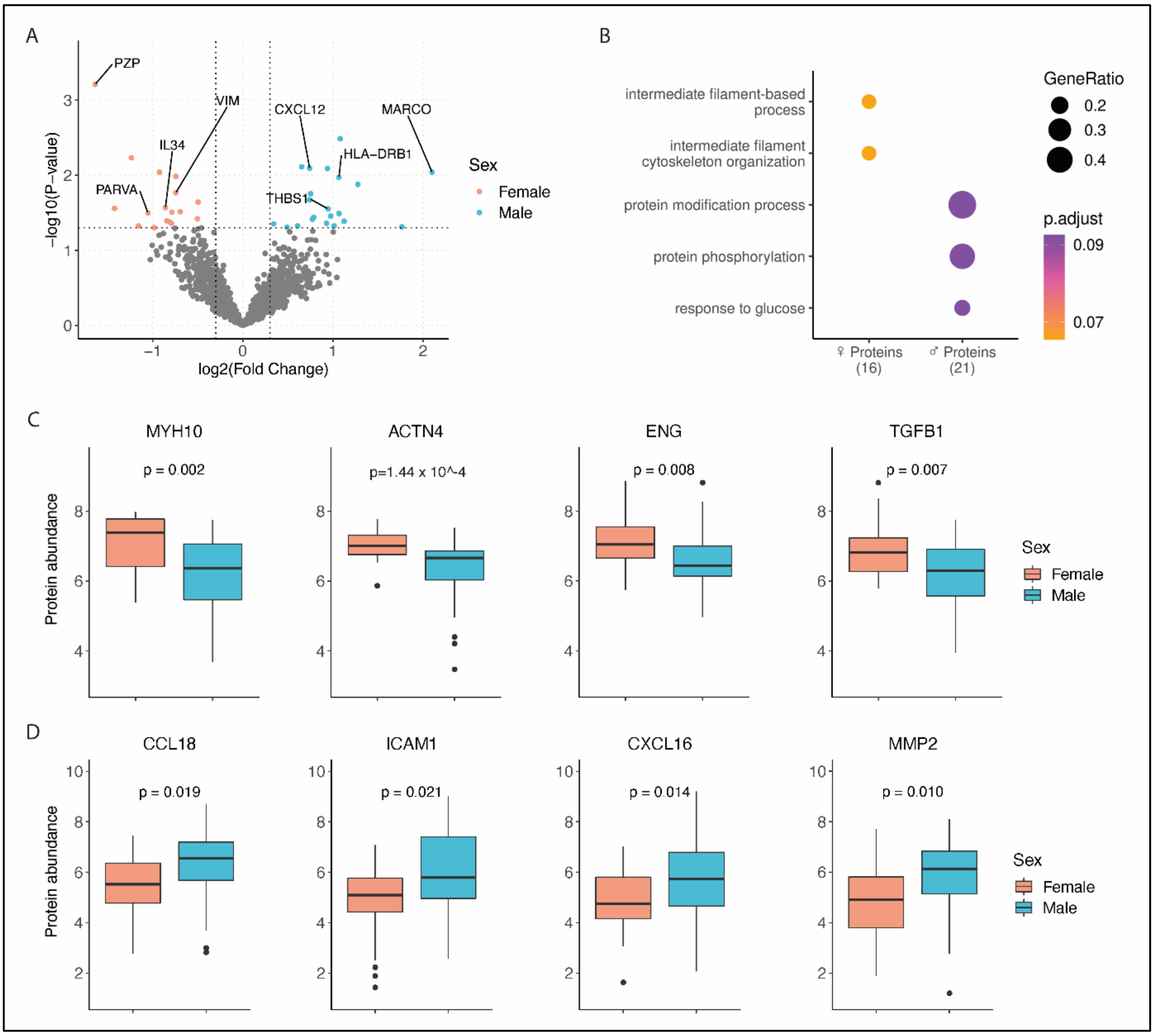

Supplemental Figure 3: Proteomic differences in abundance between male and female plaques. *A volcano plot is shown for the differential protein abundance analysis between atheromatous plaques from female and male patients (**A**). GO enrichment analyses for female-biased (left panel **B**) and male-biased proteins in atheromatous plaques (right panel **B**). Boxplots representing key female-biased (**C**) and male-biased proteins in the fibrous plaque comparison (**D**). Statistical tests: DESeq2 Negative binomial GLM + Wald test (**A**), Hypergeometric test, BH-FDR (**B**), and Two-tailed unpaired t-test (**C** & **D**).*

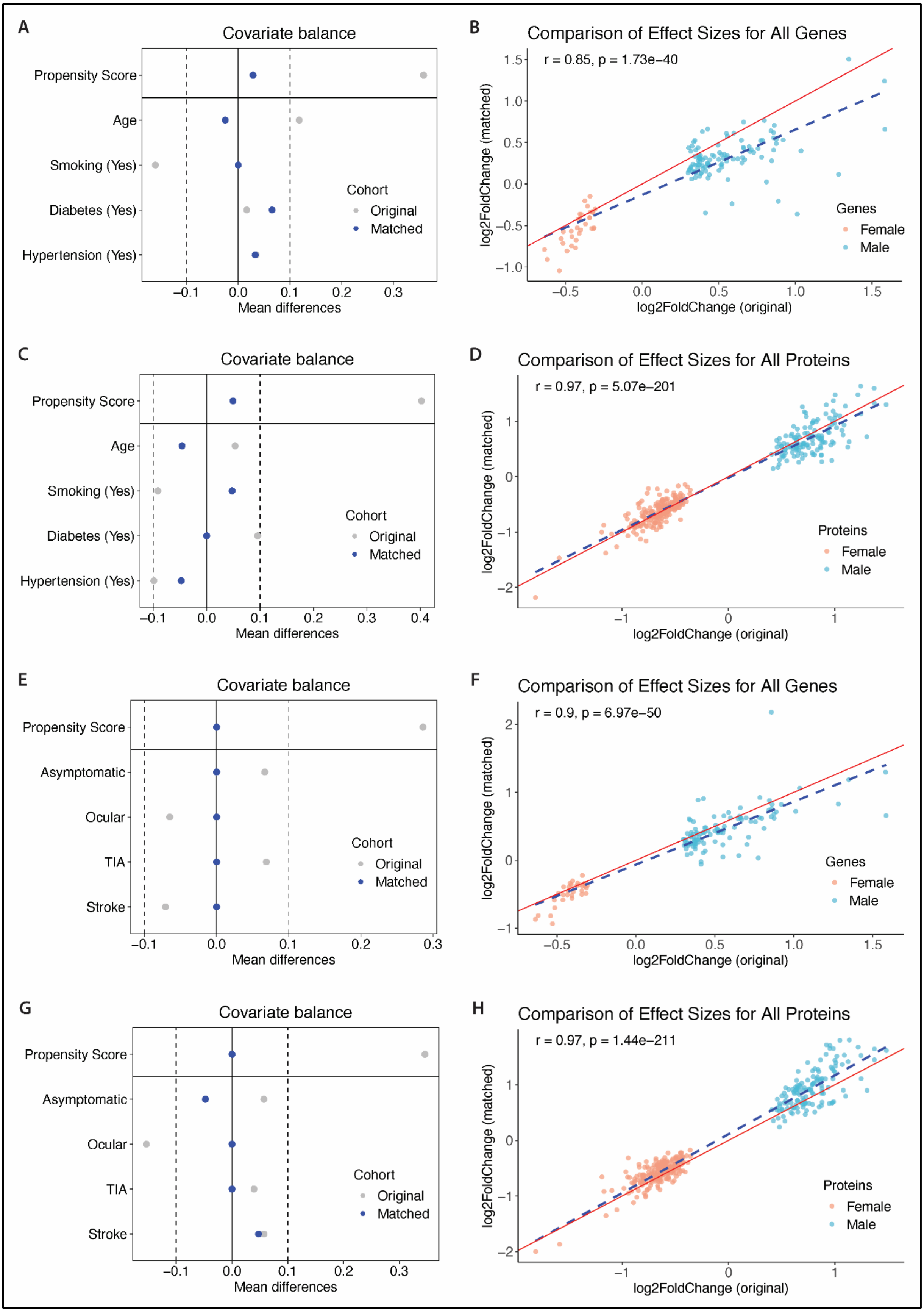

Supplemental Figure 4: Sensitivity analysis for propensity matched patients in genes and proteins. *Covariate balance before and after propensity score matching based on classical risk factors (**A** & **C**) and clinical presentation (**E** & **G**), comparing the mean differences for key covariates in the original cohort (gray) and the*

*matched cohort (blue). Vertical dashed lines indicate the threshold for acceptable balance (±0.1). Dotplots representing correlations between effect sizes for all identified genes (**B** & **F**) and proteins (**D** & **H**) between the original and matched cohort for both men (blue) and women (pink). Blue dashed line represents regression line and the red line represent the line of identity.* Statistical test: Pearson correlation (**B**, **D**, **F** & **H**).

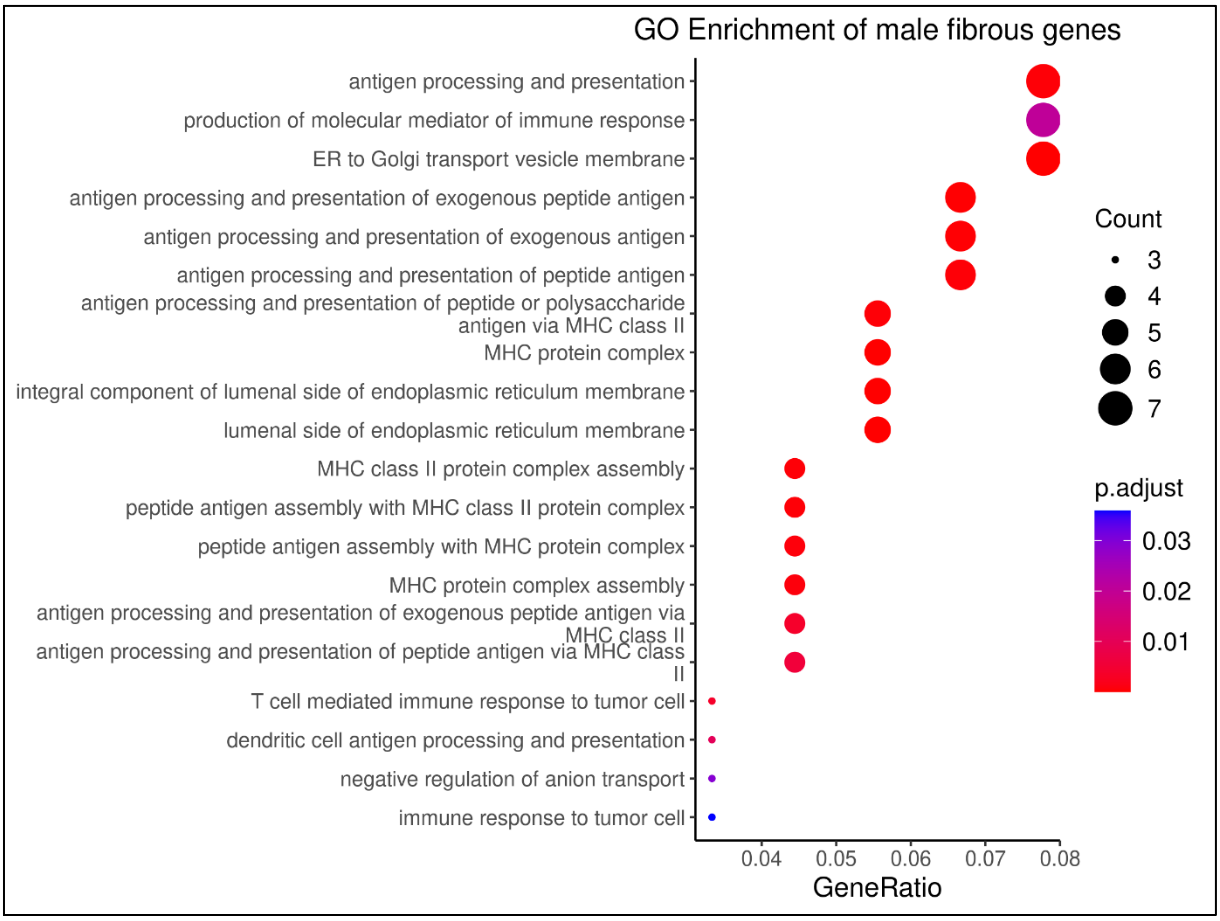

Supplemental Figure 5: GO enrichment analyses for male-biased genes in fibrous plaques from the original cohort, adjusted for risk factors (Age, Hypertension and Smoking) except Diabetes. *Statistical test: Hypergeometric test, BH-FDR.*

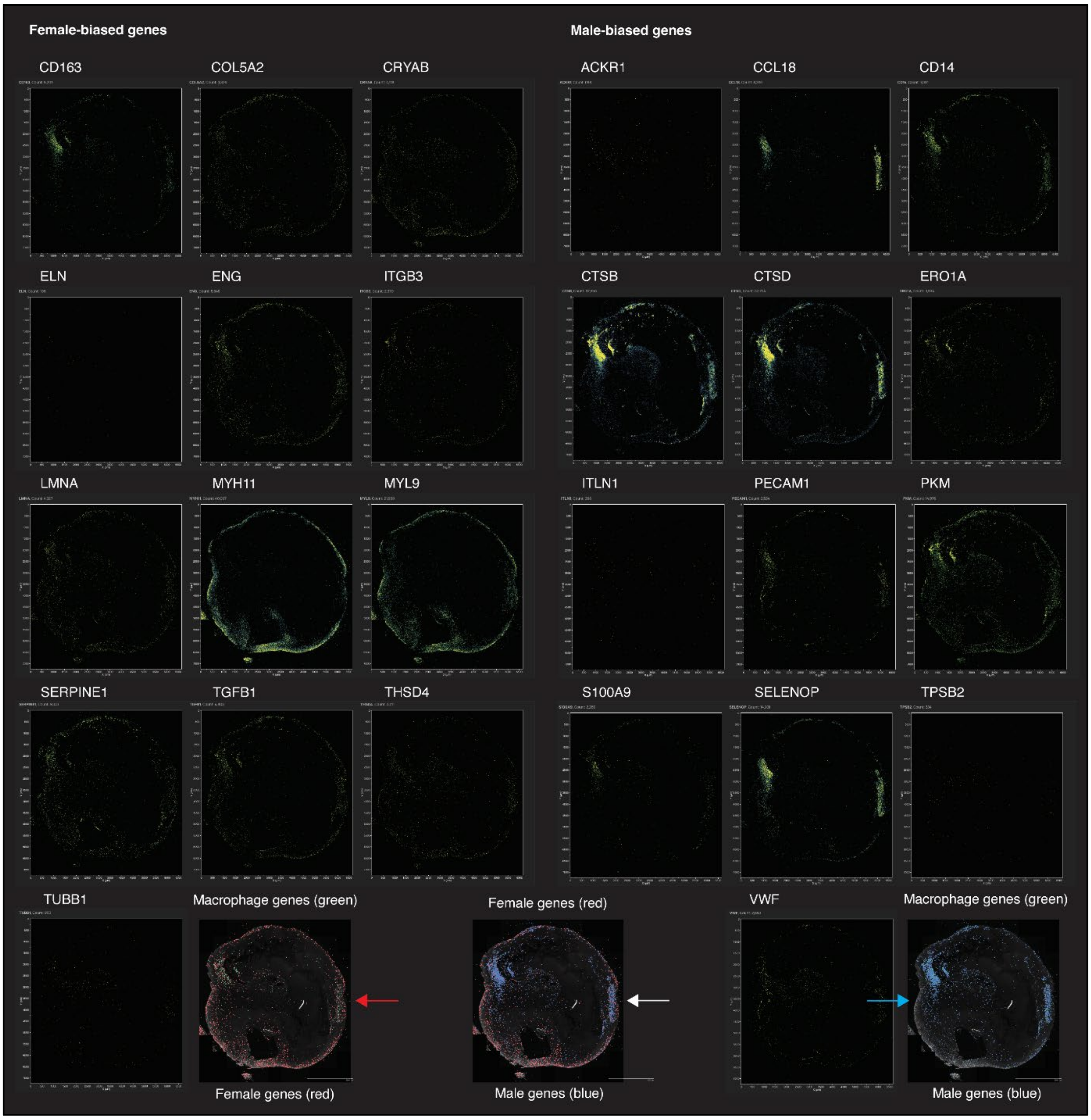

Supplemental Figure 6: Spatial transcriptomics of main sex-differential genes in a female carotid plaque. *Density plots representing the spatial distribution of female-biased genes (left, n=13) and male-biased genes (right, n=13) separately in a female plaque. Localization of female-biased genes (red arrow - bottom left; red dots) and male-biased genes (blue arrow - bottom right; blue dots) relative to classical macrophage genes (CD68, MACRO, SIRPA) (green dots in both). Localization of female-biased genes (white arrow – bottom; red dots) relative to male-biased genes (white arrow – bottom; blue dots).*

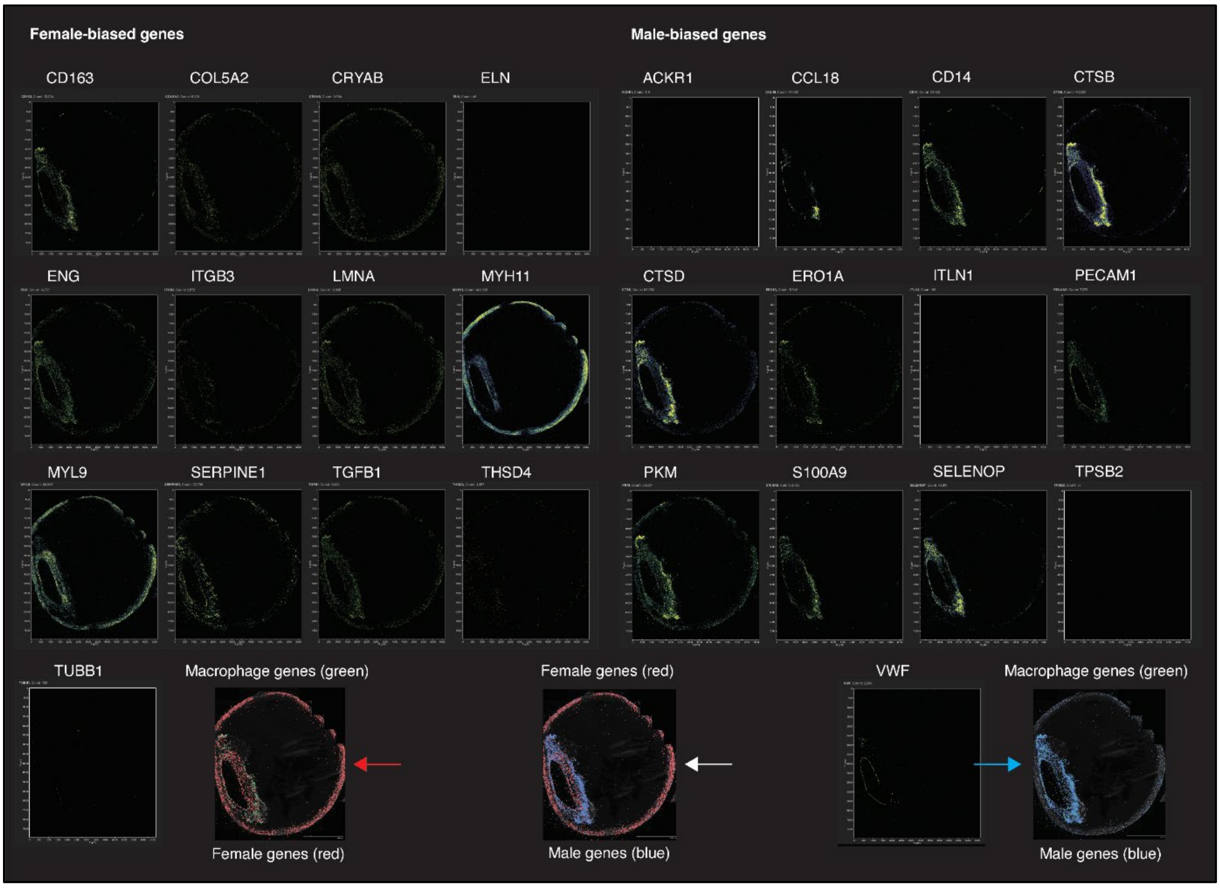

Supplemental Figure 7: Spatial transcriptomics of main sex-differential genes in a male carotid plaque. *Density plots representing the spatial distribution of female-biased genes (left, n=13) and male-biased genes (right, n=13) separately in a male plaque. Localization of female-biased genes (red arrow - bottom left; red dots) and male-biased genes (blue arrow - bottom right; blue dots) relative to classical macrophage genes (CD68, MACRO, SIRPA) (green dots in both). Localization of female-biased genes (white arrow – bottom; red dots) relative to male-biased genes (white arrow – bottom; blue dots).*

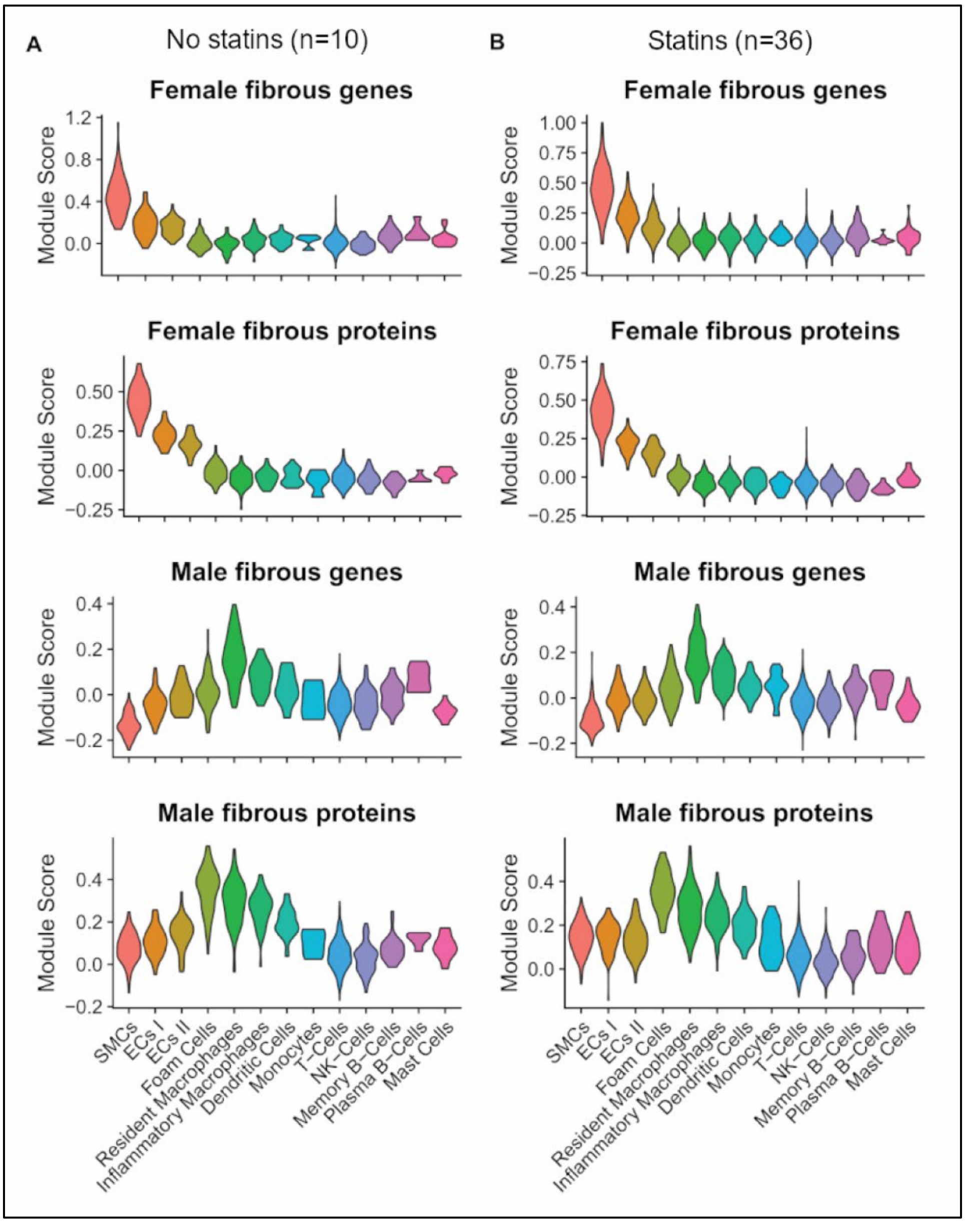

Supplemental Figure 8: Projection of module scores from female- and male-biased genes and proteins into single-cell RNAseq stratified by statins*. Module score (see Methods) expression of female fibrous genes and proteins (upper two panels* ***A****) and male fibrous genes and proteins in plaque cell types (lower two panels* ***A****) for patients who reported not taking statins at the time of the carotid endarterectomy. Module score (see Methods) expression of female fibrous genes and proteins (upper two panels* ***B****) and male fibrous genes and proteins in plaque cell types (lower two panels* ***B****) for patients who reported taking statins at the time of the carotid endarterectomy.*

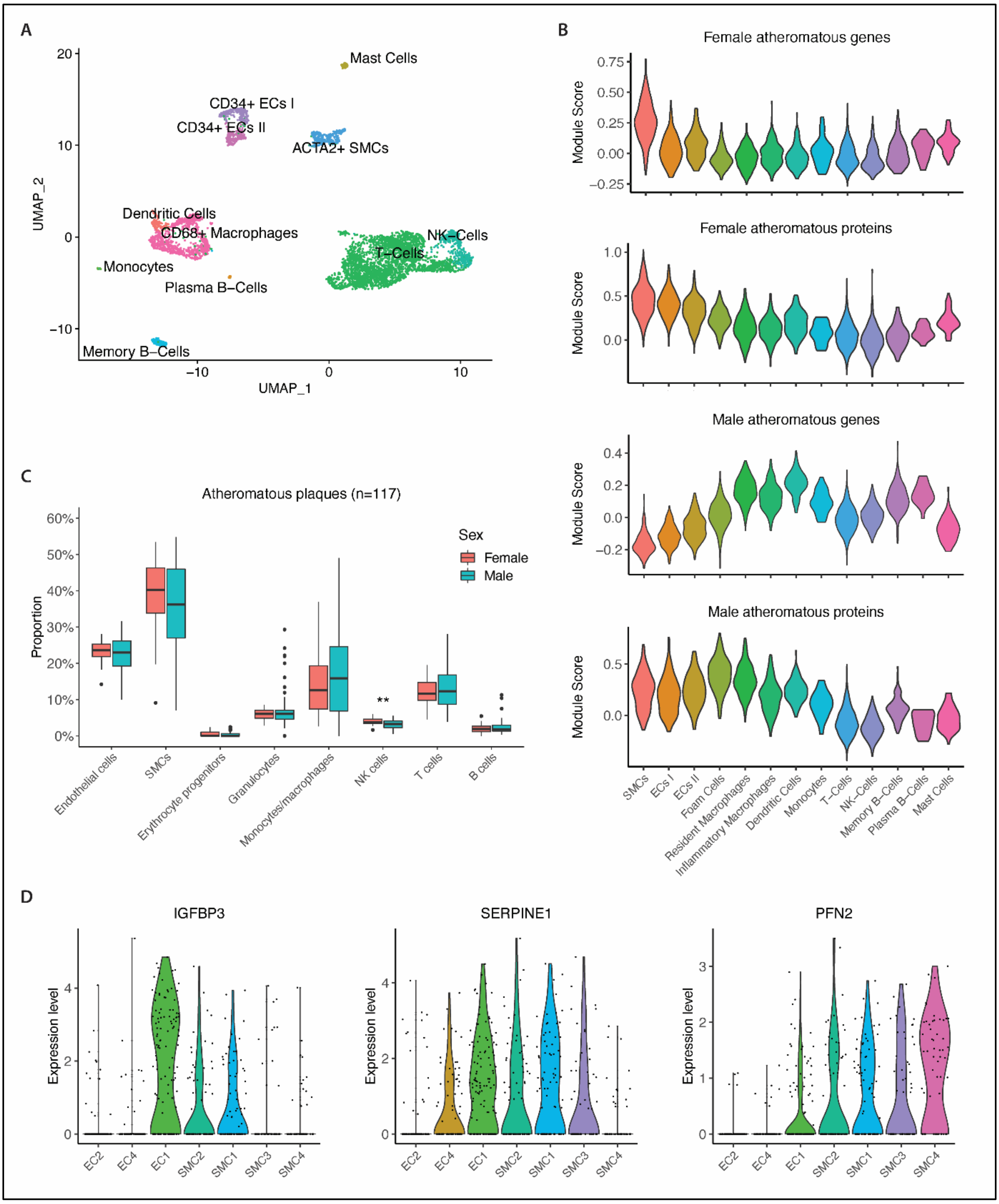

Supplemental Figure 9: Single-cell and DNA methylation deconvolution of female- and male-biased genes and proteins in atheromatous plaques. *UMAP plot of 4,948 single cells from carotid plaques (20 female and 26 male patients) (**A**). Module score (see Methods) expression of female atheromatous genes and proteins (upper two panels **B**) and male atheromatous genes and proteins in plaque cell types (lower two panels **B**). Boxplot is shown for the sex-stratified deconvolution analysis on atheromatous plaques using DNA methylation data. Proportion represents the predicted contribution (%) of each cell type to the atheromatous plaque (**C**). Expression of three example female fibrous genes (IGFBP3, SERPINE1 and PFN2) in single-cell populations of plaque ECs and SMCs (**D**). Statistical test: Two-tailed unpaired t-test (**C**).*

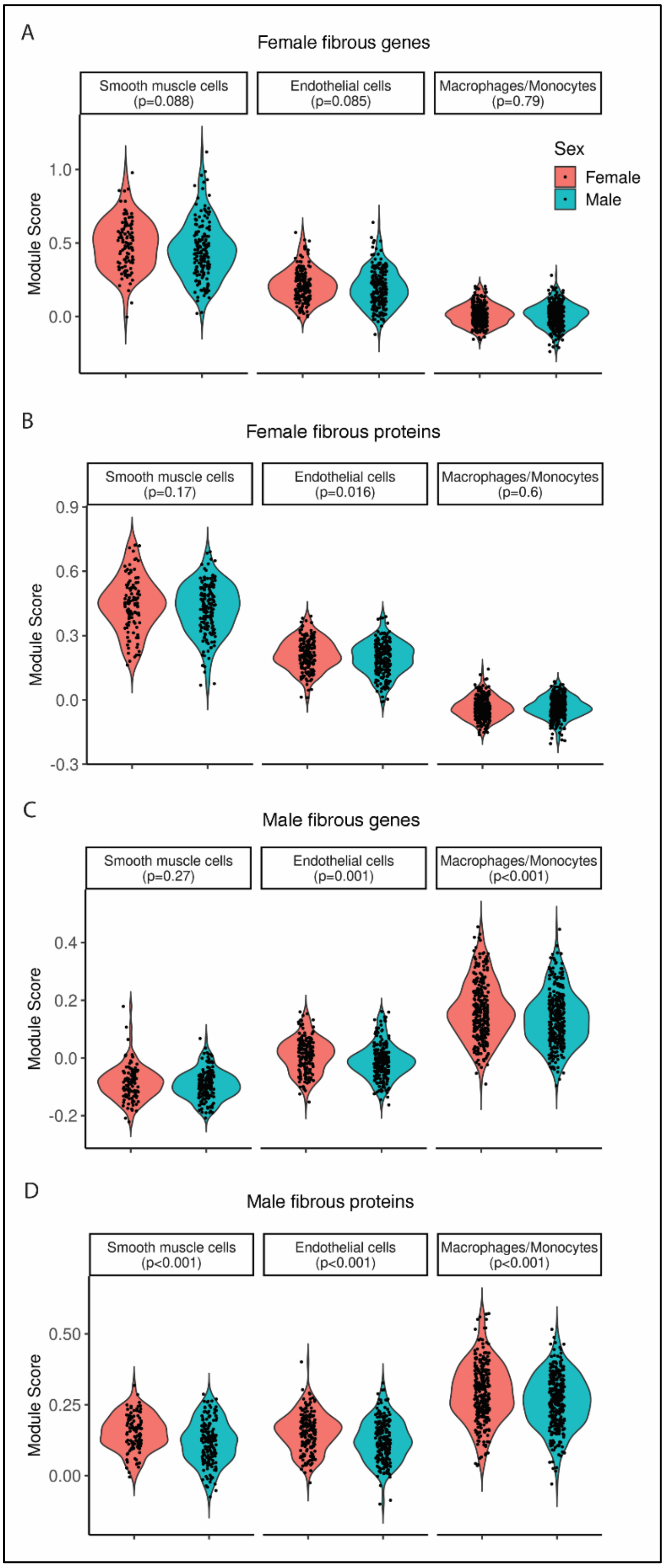

Supplemental Figure 10: Expression of genes and protein of interest by cell type in atherosclerotic plaques single cells: *Module score (see Methods) expression of female fibrous genes in main cell types of interest stratified by sex (**A**). Module score (see Methods) expression of female fibrous proteins in main cell types of interest stratified by sex (**B**). Module score (see Methods) expression of male fibrous genes in main cell types of interest stratified by sex (**C**). Module score (see Methods) expression of male fibrous proteins in main cell types of interest stratified by sex (**D**).*

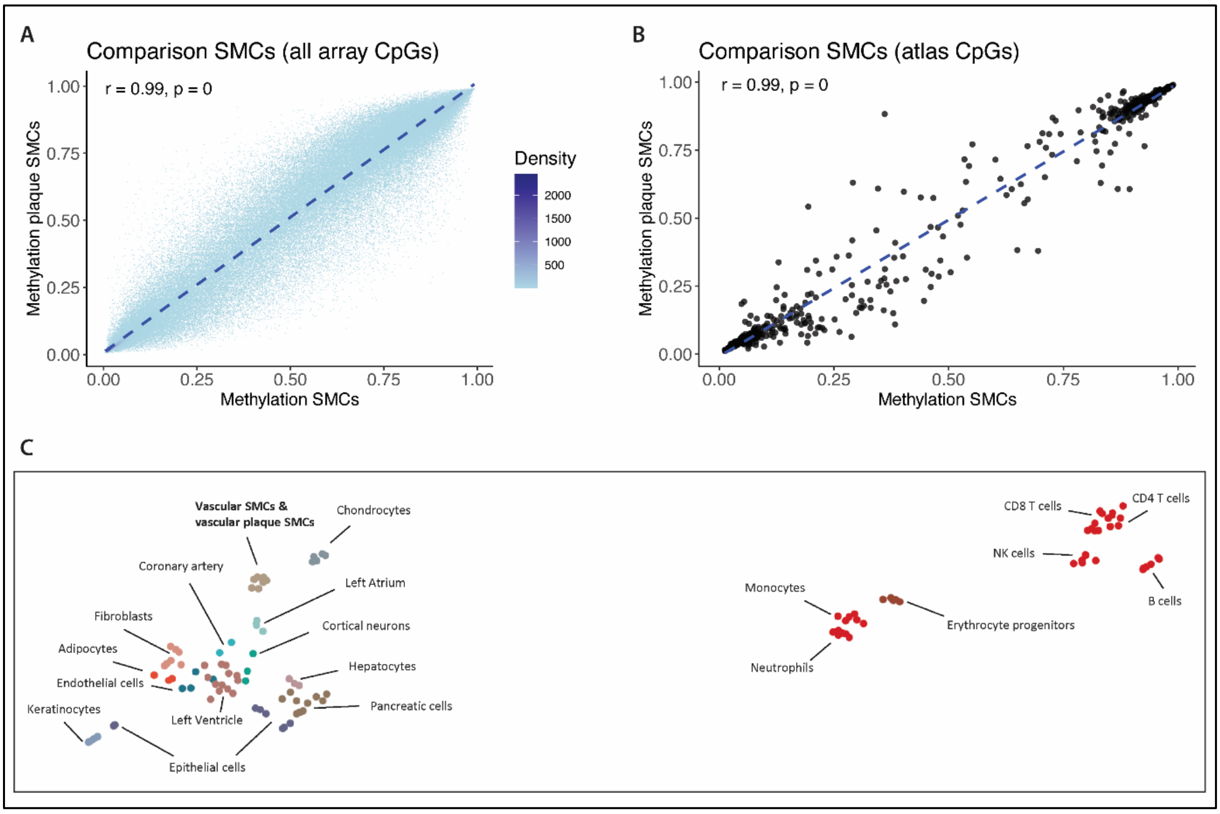

Supplemental Figure 11: Comparison tissues used in the DNA methylation deconvolution atlas including a comparison of healthy and plaque (diseased) SMCs. *Correlation of methylation levels between healthy SMCs and SMCs from plaques across all CpGs on the array (**A**). Correlation of methylation levels between healthy SMCs and SMCs from plaques across CpGs included in the methylation atlas (**B**). UMAP visualization of human cell types from a comprehensive methylation atlas (**C**).* Statistical test: Pearson correlation (**A** & **B**).

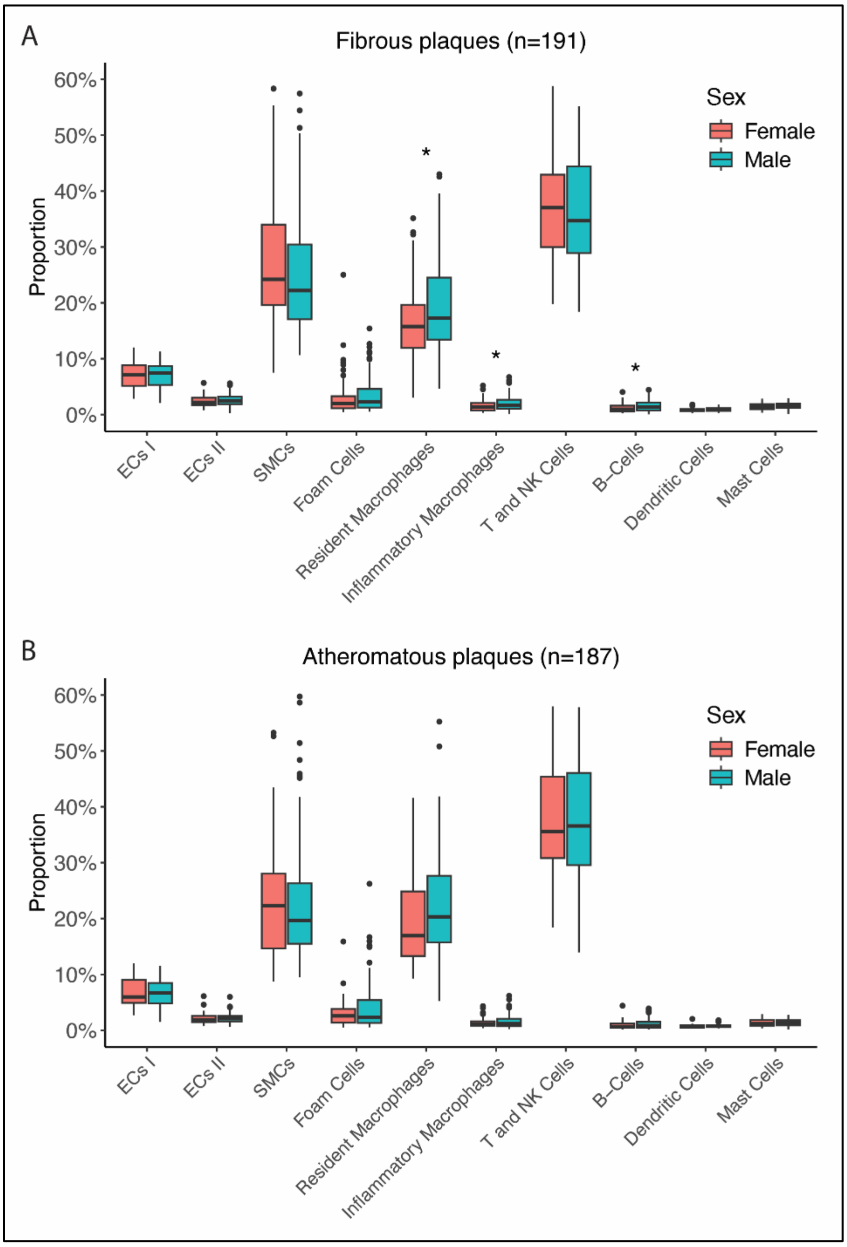

Supplemental Figure 12: Deconvolution of bulk RNAseq using scRNAseq-defined clusters as a reference. *Boxplot are shown for the sex-stratified deconvolution analysis on fibrous (**A**) and atheromatous plaques (**B**) using bulk RNAseq data. Proportion represents the predicted contribution (%) of each cell type to the plaque. Statistical test: Two-tailed unpaired t-test (**A** & **B**).*

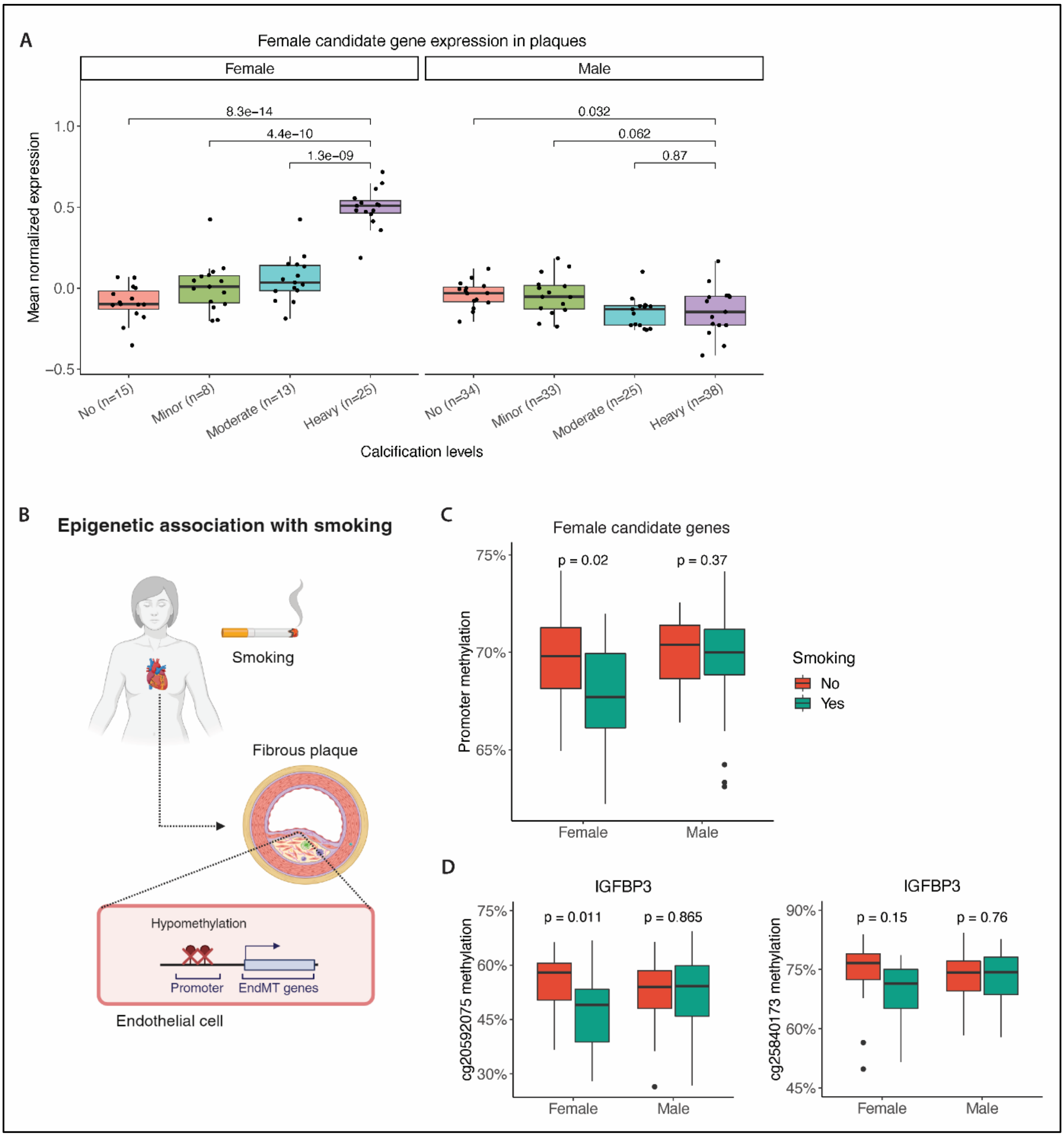

Supplemental Figure 13: Female candidate genes expression in calcified and non-calcified plaques and methylation analysis in promoters of the female candidate genes. *Boxplots showing the mean normalized expression of the female candidate genes (n=15) across plaques with increasing calcification levels, stratified by sex (**A**). Overview of the potential relation between smoking and EndMT promoter methylation changes in fibrous plaques from women (**B**). Boxplots representing the promoter methylation changes of female candidate genes (linked to CpGs in their promoters, n=4) in fibrous plaques (**C**). Boxplots representing the promoter methylation changes of female candidate gene IGFBP3 in fibrous plaques (**D**). Statistical test: Two-tailed unpaired t-test (**A**, **C** & **D**).*

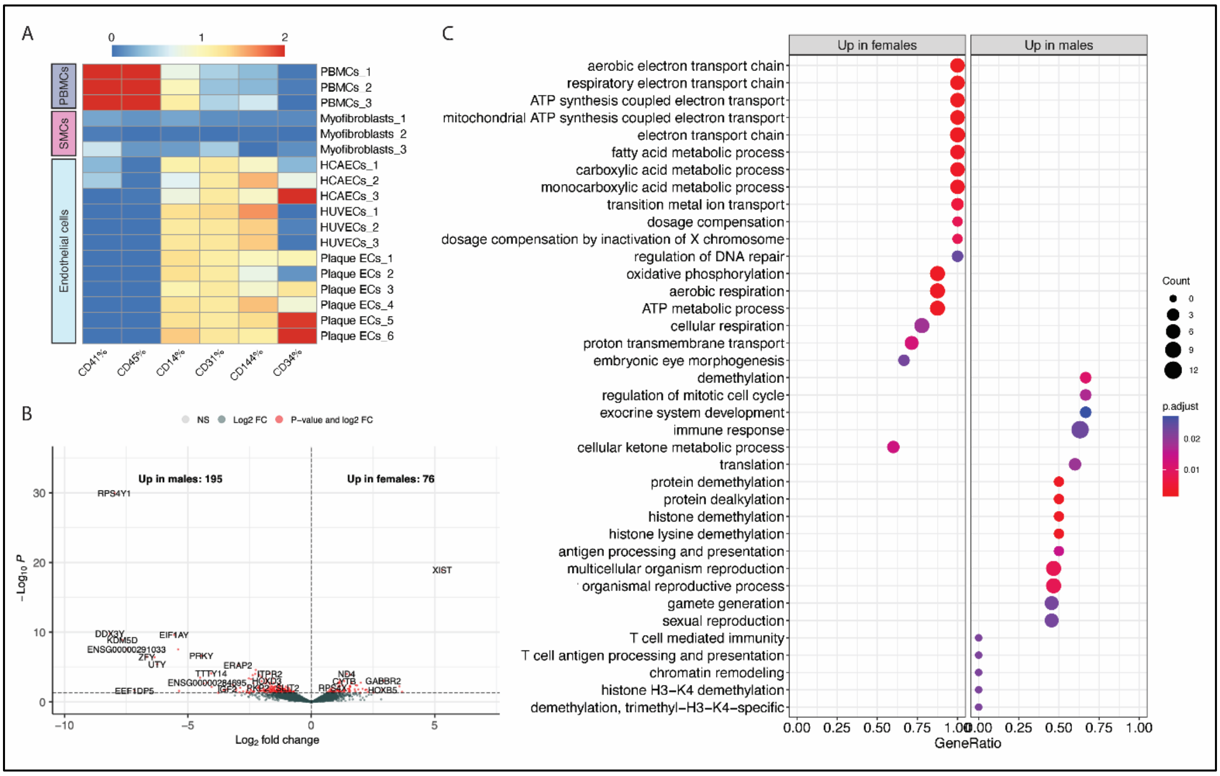

Supplemental Figure 14: Plaque EC characterization using FACS and bulk RNA-seq. *Heatmap displaying the percentage of cells positive for specific cell surface markers (columns) across various cell types (rows) using FACS. The color intensity reflects scaled percentages (**A**). A volcano plot is shown for the differential gene expression analysis between female and male plaque ECs at passage 2 (**B**). GO enrichment analysis for female-biased (left panel **C**) and male-biased genes (right panel **C**). PBMCs = peripheral blood mononuclear cells, HCAECs = human coronary artery endothelial cells, HUVECs = human umbilical vein endothelial cells. Statistical tests: DESeq2 Negative binomial GLM + Wald test (**B**), and Hypergeometric test, BH-FDR (**C**).*

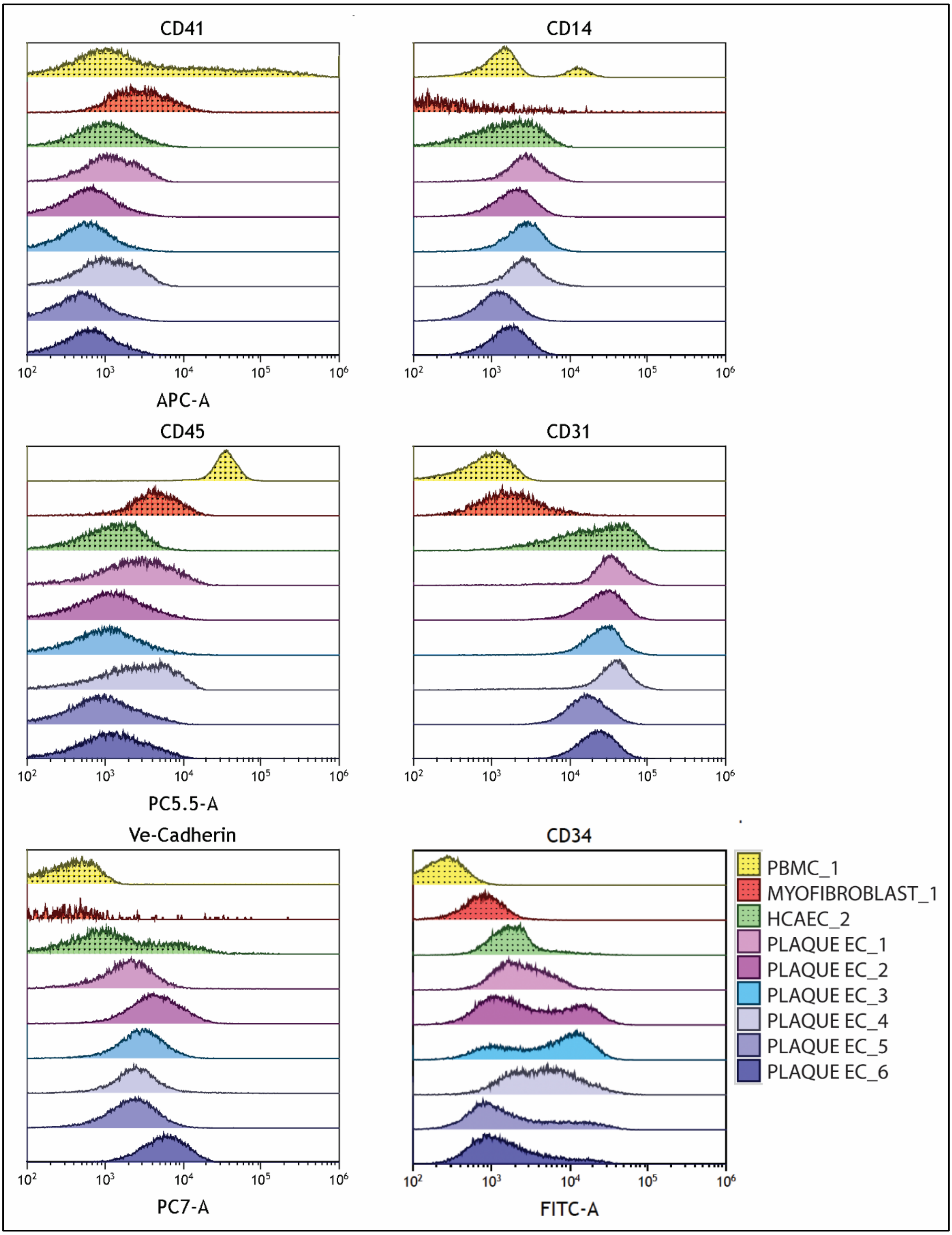

Supplemental Figure 15: Flow cytometry analysis of isolated human plaque carotid endothelial cells compared to other cell types. *Histograms representing the fluorescence intensity of cell surface markers from flow cytometry analysis across different cell types. PBMCs = peripheral blood mononuclear cells, HCAECs = human coronary artery endothelial cells.*

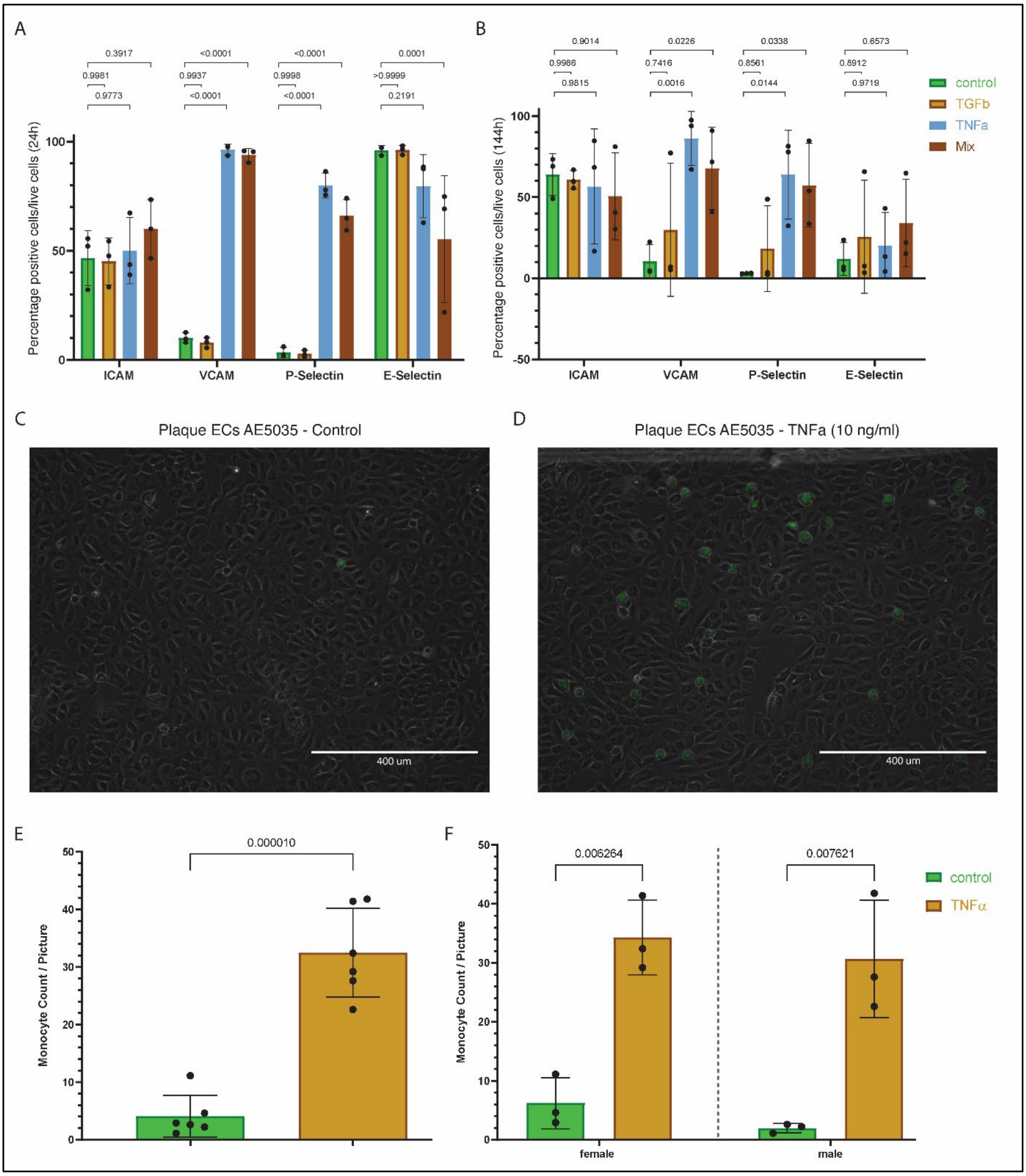

Supplemental Figure 16: Functional characterization of the activation state of plaque-derived ECs. *Barplots representing the percentage of adhesion molecule–positive cells (of live cells) from flow cytometry analysis upon different stimulations at 24h (**A**) and 144h (**B**). Representative images of monocyte adhesion under flow to plaque-derived ECs after 5h TNFα stimulation (**D**) compared with control (**C**) (green = adherent monocytes). Quantification of monocyte adhesion under flow after 5h of TNFα stimulation compared with control (**E**) and stratified by sex (**F**). Bars represent the mean, with whiskers indicating the standard deviation. Statistical test: Two-tailed unpaired t-test (**A**, **B**, **E** & **F**).*

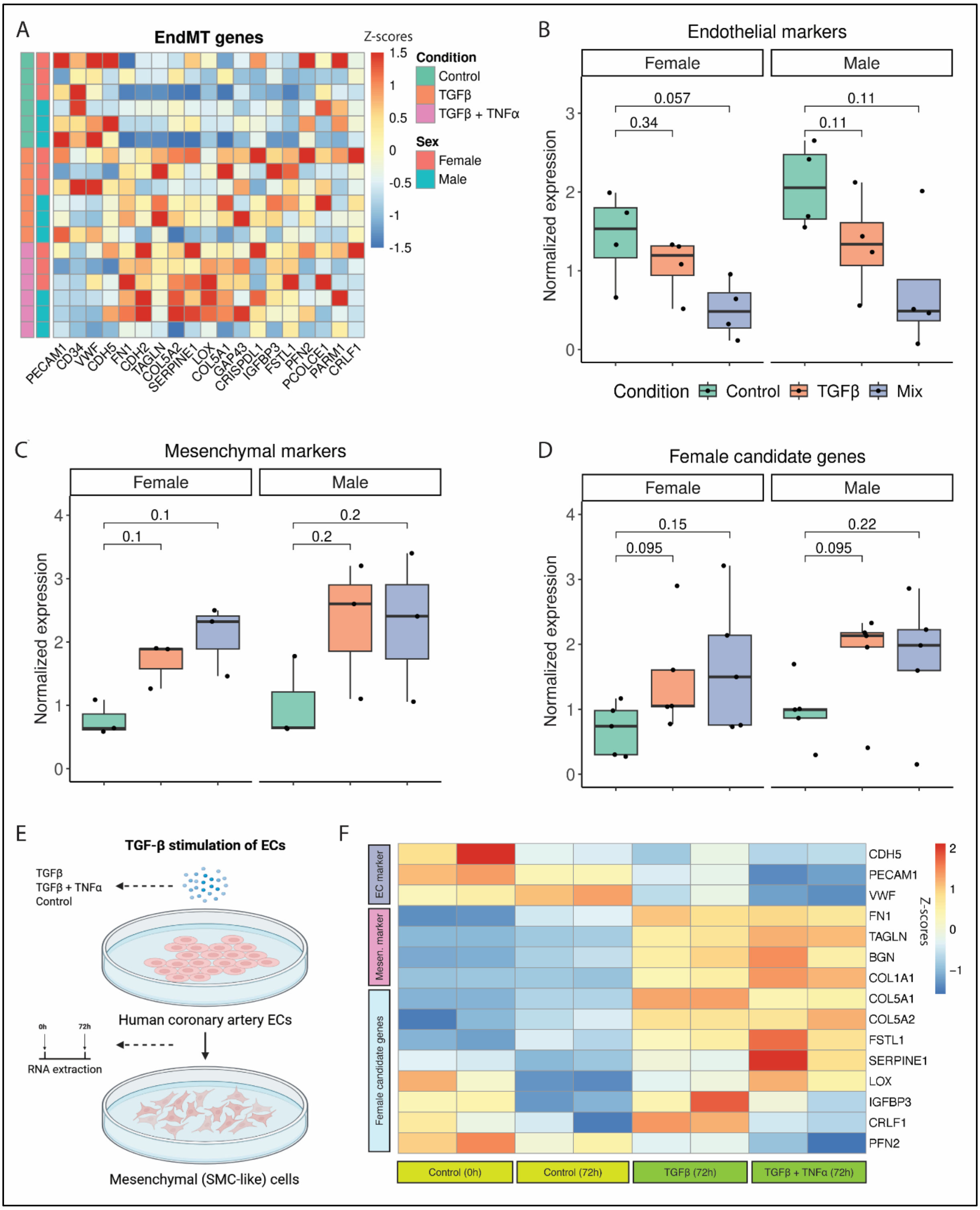

Supplemental Figure 17: TGF-β stimulation of human primary plaque ECs and coronary artery ECs in vitro. *Heatmap displaying changes in qPCR expression of known endothelial and mesenchymal markers, alongside the female candidate genes (columns), across different conditions and sexes (rows) (**A**). Boxplots showing changes in normalized qPCR expression of known endothelial markers (**B**) and mesenchymal markers (**C**), alongside the female candidate genes (**D**) compared to control (=no stimulation), across different conditions and stratified by sex. Experimental design for TGF-β stimulation of human coronary ECs (see Methods) (**E**). Heatmap displaying changes in qPCR expression of known endothelial and mesenchymal markers, alongside our female candidate genes (rows), across different conditions (columns) (**F**). Heatmaps display row-scaled Z-scores (per gene), with values standardized across conditions. Statistical test: two-tailed unpaired Mann-Whitney U test (**B**-**D**).*

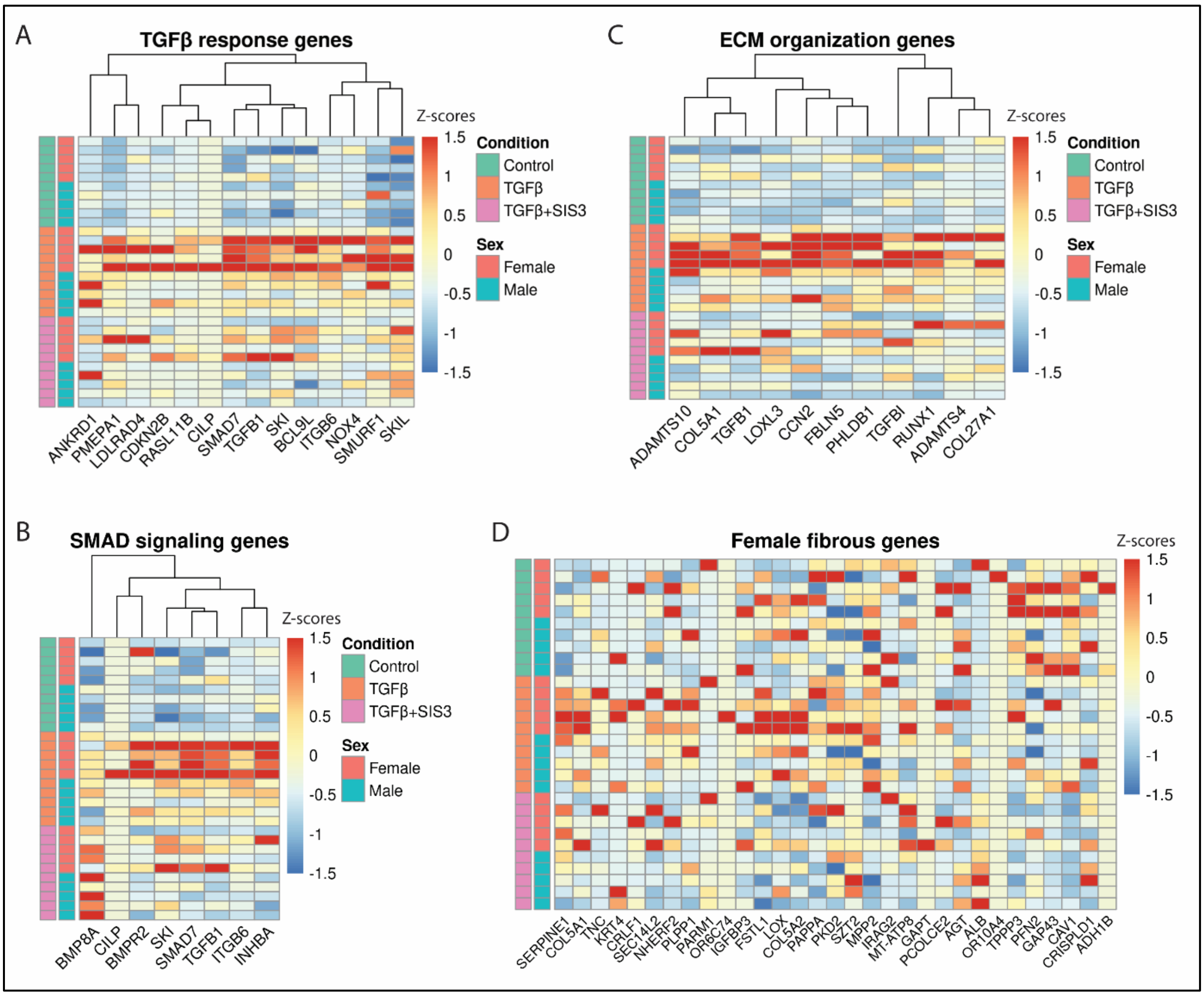

Supplemental Figure 18: TGF-β stimulation, with or without SMAD3 inhibition by SIS3, of human primary vascular SMCs in vitro. *Heatmap displaying changes in bulk RNA expression of TGF-β response genes (**A**), SMAD signaling genes (**B**), ECM organization genes (**C**), and female fibrous genes (**D**) across different conditions and sexes. Heatmaps display row-scaled Z-scores (per gene), with values standardized across conditions.*

Major Resources Table

In order to allow validation and replication of experiments, all essential research materials listed in the Methods should be included in the Major Resources Table below. Authors are encouraged to use public repositories for protocols, data, code, and other materials and provide persistent identifiers and/or links to repositories when available. Authors may add or delete rows as needed.

Animals (in vivo studies)

Species	Vendor or Source	Background Strain	Sex	Persistent ID / URL

Genetically Modified Animals

	Species	Vendor or Source	Background Strain	Other Information	Persistent ID / URL
**Parent - Male**					
**Parent - Female**					

Antibodies

Target antigen	Vendor or Source	Catalog #	Working concentration	Lot # (preferred but not required)	Persistent ID / URL
von Willebrand factor (vWF)	DAKO	A0082	1:300		Monoclonal Mouse
Ve-Cadherin	Santa Cruz Technologies	SC-9989	0.5 µg/ml		VE-cadherin Antibody (F-8) | SCBT - Santa Cruz Biotechnology
Goat anti Mouse AF488	Fisher Scientific	A11001	5 µg/ml		Goat anti-Mouse IgG (H+L) Cross-Adsorbed, Alexa Fluor™ 488 (A-11001)
Goat anti Rabbit AF555	Fisher Scientific	A21428	5 µg/ml		Goat anti-Rabbit IgG (H+L) Cross-Adsorbed, Alexa Fluor™ 555 (A-21428)

HOECHST	ThermoFischer	H1399	10 µg/ml		DAPI and Hoechst Nucleic Acid Stains 10 mL | Buy Online | Invitrogen™ | thermofisher.com
PECAM/CD31	Santa Cruz Technologies	SC-11506-R	0.5 µg/ml	C2112	Datasheet Blank Template
CD34	Bio-Rad	MCA1578F	2.5 µg/ml	15110	human-cd34-antibody-581-mca1578f.pdf
PECAM1/CD31	Biolegend	303121	1.25 µg/ml	B426324	Brilliant Violet 605 anti-human CD31 Antibody anti-CD31 - WM59
Ve-Cadherin /CD144	Biolegend	348516	5 µg/ml	B396636	PE/Cyanine7 anti-human CD144 (VE-Cadherin) Antibody anti-CD144 - BV9
CD14	Biolegend	301839	2.5 µg/ml	B454500	Brilliant Violet 785 anti-human CD14 Antibody anti-CD14 - M5E2
CD45	BD	564105	2.5 µg/ml	3156296	PerCP-Cy™5.5 Mouse Anti-Human CD45
CD41	Biolegend	303726	1.25 µg/ml	B380631	Alexa Fluor 647 anti-human CD41 Antibody anti-CD41 - HIP8
Zombie NIR	Biolegend	77184	100 tests	B390466	Zombie NIR Fixable Viability Kit
ICAM	Biolegend	322713	10 µg/ml	B461130	Alexa Fluor 488 anti-human CD54 Antibody anti-CD54 - HCD54
VCAM	Biolegend	305815	1.25 µg/ml	B440993	Brilliant Violet 421™ anti-human CD106 Antibody, CD106, STA
P-selectin	Biolegend	304941	2.5 µg/ml	B409408	Brilliant Violet 785™ anti-human CD62P (P-Selectin) Antibody, CD62P, AK4
E-selectin	Biolegend	336015	10 µg/ml	B461096	PE/Cyanine7 anti-human CD62E Antibody, CD62E, HAE-1f
Tissue Factor/ CD142	Biolegend	365203	1.25 µg/ml	B445781	PE anti-human CD142 Antibody anti-CD142 - NY2

DNA/cDNA Clones

Clone Name	Sequence	Source / Repository	Persistent ID / URL

Cultured Cells

Name	Vendor or Source	Sex (F, M, or unknown)	Persistent ID / URL
HCAECs	Promocell CAT#C-12221	Female	Human Coronary Artery Endothelial Cells (HCAEC) | PromoCell
HCAECs	Promocell CAT#C-12221	Female	Human Coronary Artery Endothelial Cells (HCAEC) | PromoCell
HCAECs	Promocell CAT#C-12221	Male	Human Coronary Artery Endothelial Cells (HCAEC) | PromoCell
Plaque myofibroblasts	UMC	Male	Human Plaque Myofibroblasts to Study Mechanisms of Atherosclerosis - PubMed
HUVEC	UMC	Female	
PBMCs	UMC, (Mini Donor Service)	Female	
THP-1 monocytes	ATCC	Unknown	TIB-202; THP-1 - TIB-202 | ATCC
Aortic SMCs Donor 1	UCLA	Female	
Aortic SMCs Donor 2	UCLA	Female	
Aortic SMCs Donor 3	UCLA	Female	
Aortic SMCs Donor 4	UCLA	Female	
Aortic SMCs Donor 5	UCLA	Female	
Aortic SMCs Donor 6	UCLA	Male	
Aortic SMCs Donor 7	UCLA	Male	
Aortic SMCs Donor 8	UCLA	Male	
Aortic SMCs Donor 9	UCLA	Male	
Aortic SMCs Donor 10	UCLA	Male	
Plaque ECs Donor 1	UMC	Male	

Plaque ECs Donor 2	UMC	Male	
Plaque ECs Donor 3	UMC	Female	
Plaque ECs Donor 4	UMC	Male	
Plaque ECs Donor 5	UMC	Female	
Plaque ECs Donor 6	UMC	Female	
Plaque ECs Donor 7	UMC	Male	
Plaque ECs Donor 8	UMC	Male	
Plaque ECs Donor 9	UMC	Female	
Plaque ECs Donor 10	UMC	Female	
Plaque ECs Donor 11	UMC	Male	
Plaque ECs Donor 12	UMC	Male	
Plaque ECs Donor 13	UMC	Male	
Plaque ECs Donor 14	UMC	Male	
Plaque ECs Donor 15	UMC	Male	
Plaque ECs Donor 16	UMC	Female	
Plaque ECs Donor 17	UMC	Male	
Plaqye ECs Donor 18	UMC	Female	

Data & Code Availability

Description	Source / Repository	Persistent ID / URL

Other

Materials	Source / Repository	Persistent ID / URL
Animal-free Collagenase/Dispase Blend II	Merck	REF#SCR140

EBM-MV, phenol red-free	Promocell	REF#C-22225
EBM cell growth MV Kit	Promocell	REF#C-39220
Penicillin/Streptomycin	Fisher Scientific	REF#15-140-122
Primocin	Invivogen	REF#ant-pm-2
PBS	Gibco	REF#10010056
FBS	Corning	REF#35-079-CV
EDTA	ASC Reagent	REF#101448118
Charcoal stripped FBS	ThermoFischer Scientific	REF#12676029
TGF-β2	Peprotech	REF#100-35
TNFα	Miltenyi Biotec	REF#130-094-018
SIS3	Merck	REF#566405-1MG
TriPure	Roche	REF#11667165001
CellTrace CFSE Cell proliferation kit	ThermoFischer Scientific	REF#C34554
Smooth Muscle Cell Basal Medium	Lonza	REF#CC-3182
Smooth Muscle Medium-2 SingleQuots Kit	Lonza	REF#CC-4149
TGF-β1	R&D Systems	REF#7754-BH

ARRIVE GUIDELINES
The ARRIVE guidelines (https://arriveguidelines.org/) are a checklist of recommendations to improve the reporting of research involving animals. Key elements of the study design should be included below to better enable readers to scrutinize the research adequately, evaluate its methodological rigor, and reproduce the methods or findings.

Study Design

**Groups**	**Sex**	**Age**	**Number (prior to experiment)**	**Number (after termination)**	**Littermates (Yes/No)**	**Other description**
Group 1 (Control)						
Group 2						
Add more if needed						

Sample Size: Please explain how the sample size was decided Please provide details of any a *prior* sample size calculation, if done.

Inclusion Criteria

Exclusion Criteria

Randomization

Blinding
